# Empirical insights into residual emissions in urban net-zero targets

**DOI:** 10.1038/s41558-026-02691-0

**Published:** 2026-08-14

**Authors:** Giulia Ulpiani, Quirina Rodriguez Mendez, Valeria Todeschi, Gema Hernandez Moral, Nadja Vetters, Marco Pittalis, Sabine Fuss, Christiaan Gevers Deynoot, Felix Creutzig

**Affiliations:** 1 European Commission, Joint Research Centre (JRC), Ispra, Italy; 2 Potsdam Institute for Climate Impact Research (PIK), Member of the Leibniz Association, Potsdam, Germany; 3 European Commission, Joint Research Centre (JRC), Brussels, Belgium; 4 Geography Department, Humboldt University Berlin, Berlin, Germany; 5 Institute for Responsible Carbon Removal, American University, Washington, DC USA; 6 Bennett Chair for Innovation and Policy Acceleration, University of Sussex, Brighton, UK

**Keywords:** Climate-change mitigation, Carbon capture and storage

## Abstract

Cities increasingly pledge net-zero emissions targets, but the robustness of their residual emissions strategies remains uncertain. We examine the approaches of 103 European cities aiming at net zero by 2030. Total residual emissions are 61.4 MtCO_2_e originating mostly from buildings and transport, with the median at 0.8 tCO_2_e per capita, indicating a rapid decarbonization process. All municipal strategies rely on temporary, land-based CO_2_ removal, then carbon credits (40%) and permanent CO_2_ removal (32%). We develop the residual emissions strategy robustness index to assess the robustness of compensation strategies, indicating that the current maturity is medium-low with notable geographical disparities. Although cities invest in options mapping and governance, little attention is paid to land availability, the potential of the urban fabric to become a distributed carbon sink, provisions against carbon reversals, monitoring, timing and quantification (estimates cover only 18% of total residual emissions). We also distil recommendations and best practices to address residual emissions.

## Abstract (web summary)

Although many cities have set net-zero targets, there is limited understanding of their residual emissions strategies. An analysis of 103 European cities reveals best practices but also a potential over-reliance on temporary land-based carbon removal and the limited robustness of compensation strategies.

## Main

Net-zero commitments are proliferating among countries, corporations and subnational entities, covering over two-thirds of the global economy^1^ and nearly 80% of global emissions and population^2^. The scientific consensus leaves no alternative to net-zero and net-negative emissions for stabilizing global temperatures^3,4^. However, the robustness and integrity of pledged net-zero targets is very heterogeneous in terms of their timing, bindingness, coverage, governance arrangements and other desiderata, notably on carbon dioxide removal (CDR) and offsetting^1,5^.

To move forward, a growing literature calls for clear definitions and operational value around net-zero and residual (unabated) emissions at all governance levels^6–8^. It highlights the risk that net-zero imaginaries and their reliance on compensation strategies favour removals over immediate emissions reductions^1,9,10^, potentially leading to delayed or deterred action^11–13^ while incurring high costs, potential environmental consequences^14,15^, social justice implications^8,16–18^ and risk of impermanence^19,20^. However, when accompanied by risk-mitigating policies, CDR supports net-zero targets, sustainable development and ecosystem services^21,22^. CDR can enhance rural livelihoods through new income streams^23^, increased agroecosystem resilience^24^ and crop yields through soil carbon sequestration and enhanced rock weathering^25^ and reduced coastal erosion through mangrove forest restoration^26^. In urban contexts, CDR can mitigate urban heat islands^27^, enhance human health^28,29^ and promote biochar conversion of municipal waste^30,31^.

Cities, as a decision‑making layer increasingly committed to net‑zero targets, constitute a key yet under‑studied arena for CDR implementation. Early estimates show that a portfolio of urban CDR options, including vegetation, soil, built environment and decentralized direct air capture and storage (DACCS) could reach up to 1 GtCO_2_ yr^−1^ globally^32^, with additional potential contributions from alternative construction materials^33^ and bioenergy with carbon capture and storage (BECCS) from municipal waste^34^. Realizing this potential while upholding the integrity of urban climate commitments, requires careful integration of CDR into city action plans.

Here we undertake an empirical assessment of urban net-zero strategies, based on the Climate-Neutrality Action Plans (CNAPs) which 103 European cities selected under the 100 Climate-Neutral and Smart Cities Mission (hereinafter Mission Cities) submitted as a first step in their journey towards net-zero emissions by 2030. Within the Mission, residual emissions are primarily scope 1 and 2 emissions (Methods) which will remain by 2030, requiring compensation. In total, 362 European cities had expressed interest in the Mission and a preliminary investigation unveiled that (1) nearly half of those having a clear net-zero ambition could not specify the percentage of residual emissions; (2) those who could, declared residual emissions below 20%, on average, pointing at the transport sector as the greatest contributor; and (3) only 21 cities already had a clear strategy for emissions compensation^35^. We assess the progress made by 103 Mission Cities in (self) defining and addressing residual emissions in the first iteration of their CNAPs, providing insights into their magnitude, sectoral origin and strategic planning (bridging a crucial gap in literature^7,36^). Best practices from Mission Cities are presented in Supplementary Information.

## Magnitude of residual emissions

**Fig. 1:**
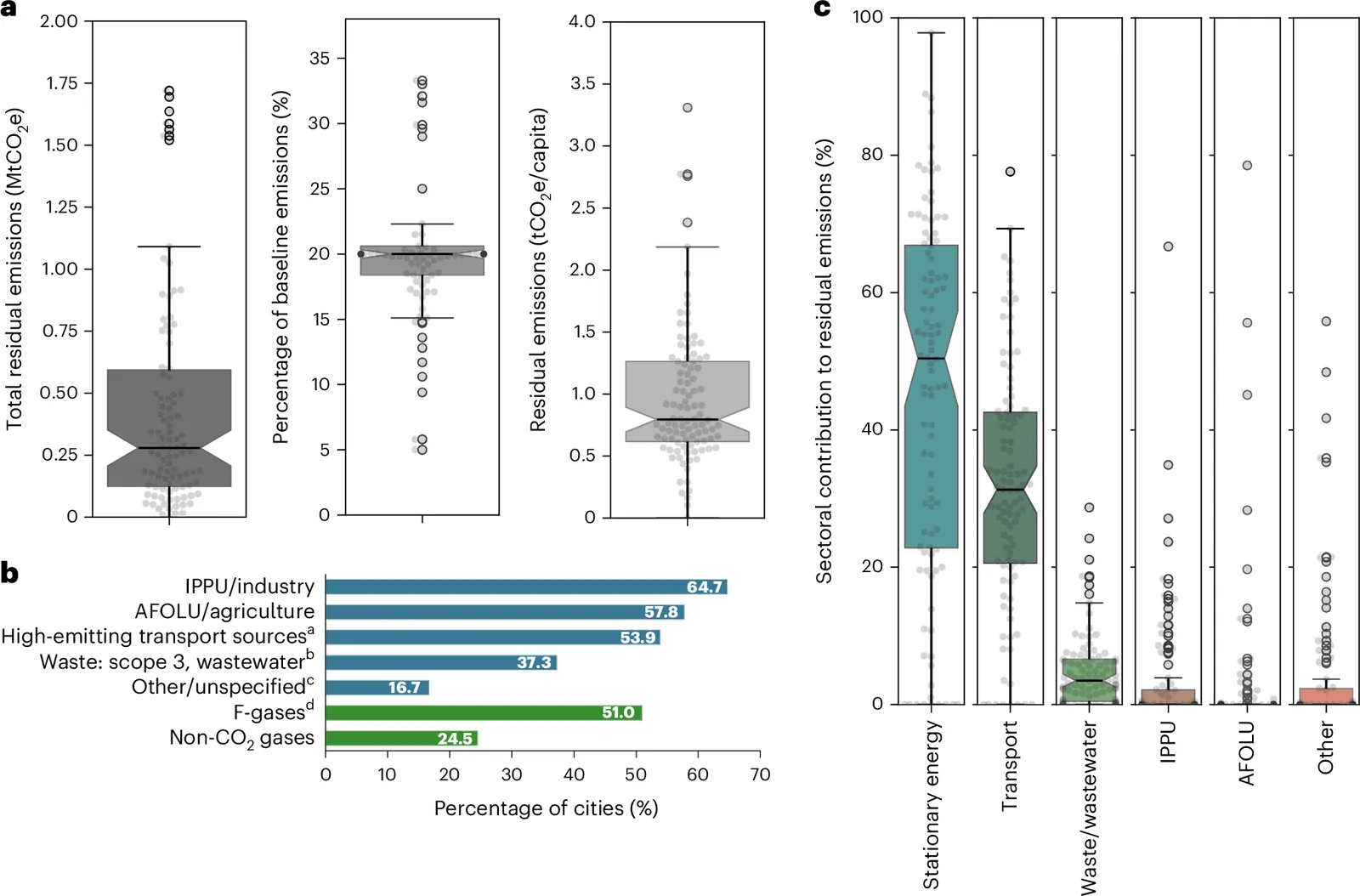
Residual emissions by 2030. **a**–**c**, Statistical distribution of total residual emissions in MtCO_2_e, their share of baseline emissions (the inventories used to set the target and quantify the associated emissions reduction in the CNAPs) and residual emissions per capita in tCO_2_e/capita (**a**) vis-à-vis the source of emissions typically excluded from the climate-neutrality target (**b**) and the percentage contribution of each accounting sector (**c**). The *y* axes of the box + swarmplots are adjusted to improve visibility and negative values are removed from **c** as they derive from accounting misconceptions in the CNAPs that cannot be corrected (six instances). The boxplots are based on the full sample of 103 cities and show the median (centre line), IQR (box; 25th–75th percentiles) and whiskers extending to 1.5× IQR. ^a^Ports/waterborne navigation, airports/aviation, highways, motorways and/or railways. ^b^Scope 3 emissions associated with waste/wastewater treatment and disposal. ^c^Geographical areas, entire scopes, specific stationary energy sources and so on. ^d^All F-gases or a subgroup. AFOLU, agriculture, forestry and other land use; F-gases, fluorinated gases; IPPU, industrial processes and product use.

The total residual emissions indicated by the 103 cities in their CNAPs is 61 MtCO_2_e, close to the emissions of Austria in 2023 (69 MtCO_2_e)^37^. The median is 279 ktCO_2_e (0.8 tCO_2_e per capita) and the interquartile range (IQR) is 124–593 ktCO_2_e (0.6−1.3 tCO_2_e/capita) (Fig. 1a). Compared with initial estimates^35^, 17 cities increased the percentage of residual emissions, 7 cities reduced it and 22 cities confirmed their ambition, so that residual emissions remain close to 20% of baseline emissions^38^ (median 20%, IQR 18–21%; Fig. 1a). When residual emissions are compared with the most recent inventories provided in the CNAPs (older than 2018 in one case versus 16% for baseline emissions), the median remains 20%, but the IQR (18–31%) leans more towards higher percentages. For 52% of the cities, residual emissions appear to be the result of target setting, with no analysis of their origin or constraints to reduction. The residual emissions of Mission Cities account for scope 3 emissions only in the waste sector and disregard sources of emissions cities often decided to exclude from the 2030 climate-neutrality target, such as fluorinated gases, industry, ports and airports (Fig. 1b; Methods).

Stationary energy (buildings, facilities, equipment, including public lighting) and transport are by far the greatest contributors to residual emissions (and baseline emissions) (Fig. 1c; Methods). Stationary energy hits the highest values (in some instances fully covering residual emissions), highest median (50%) and largest IQR (23–67%). The median share for transportation is 31% (IQR 21–43%), whereas all other sectors contribute 4% at most. There is a statistically significant correlation (*P* < 0.05, Pearson test) between the magnitude of residual emissions and the presence of ports or airports within the administrative boundary of the city, suggesting that, despite the frequent exclusion of ports and airports from the 2030 targets, their associated ancillary operations, ground logistics and induced mobility patterns may significantly contribute to persistent and difficult-to-abate urban emissions.

## Options for residual emissions compensation

**Fig. 2:**
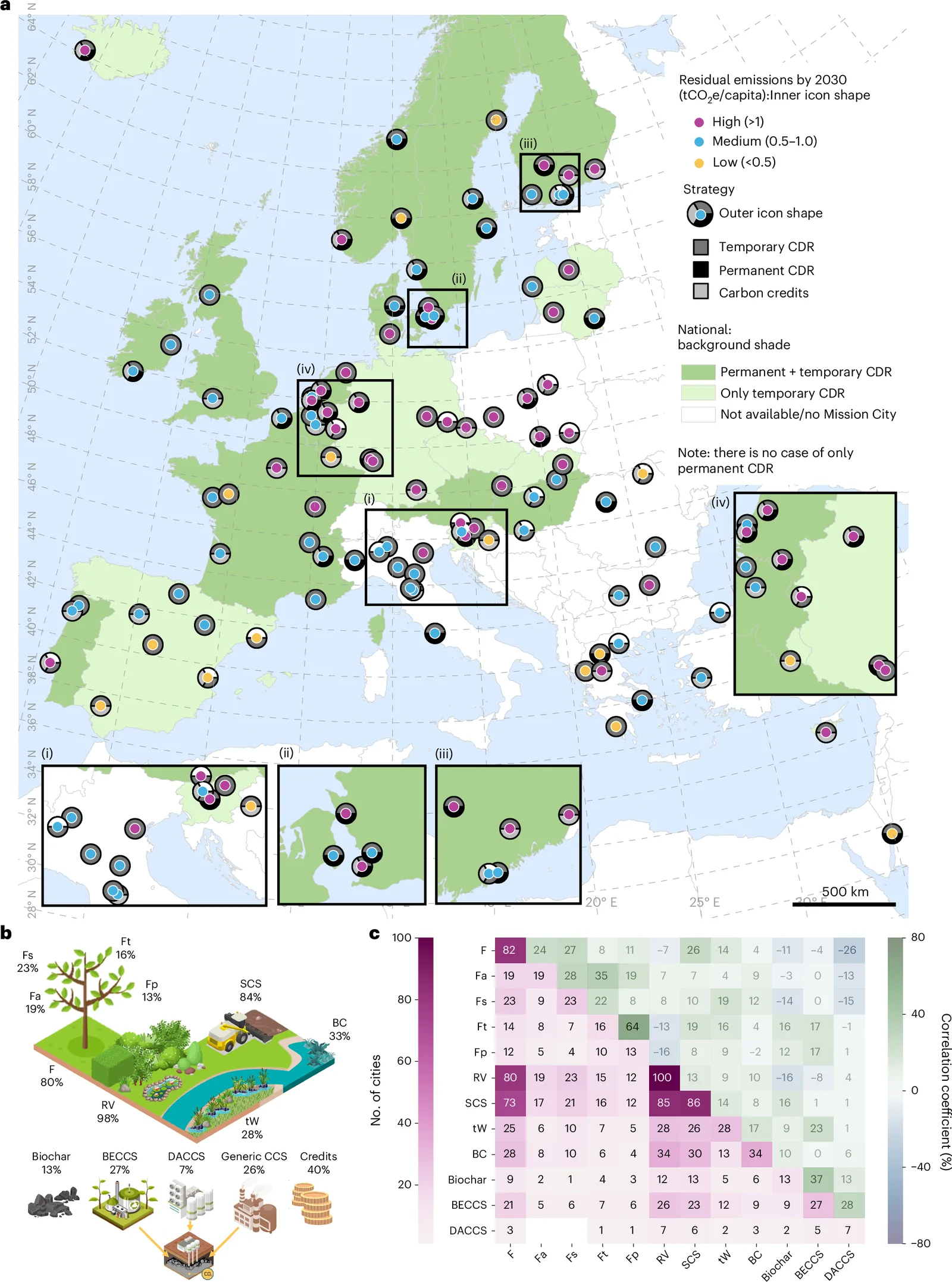
Synoptic visualization of the emissions compensation options mentioned by the 103 Mission Cities in their CNAPs. **a**, The 103 cities, with Eindhoven and Helmond co-represented at the coordinates for Eindhoven because of their joint CNAP. Each city is symbolized by a two-layer icon: a central coloured circle indicating residual emissions per capita (high >1 tCO_2_e/capita; low ≤0.5 tCO_2_e/capita; and medium 0.5–1.0 tCO_2_e/capita) and an outer ring representing the strategy of the city. The ring is divided into sectors to illustrate the combination of permanent CDR (black for atmospheric carbon and white for carbon capture with unclear application to either fossil or atmospheric carbon), temporary CDR (dark grey) and carbon credits (light grey). The colour and segmentation of the ring reflect the number of strategies used: three sectors for all three options, two sectors for two options and a single colour for one option. The background colour shows the compensation options pursued at country level. Insets (i)–(iv) enlarge dense regions of the figure to enhance its clarity. **b**, The percentage of cities that selected specific individual options as identified and coded in Methods. **c**, The combination of two heatmaps: the one located at the bottom corner shows the number of cities pursuing each combination of options, with one-option strategies represented along the diagonal; the one located at the top corner is a correlation matrix showing the correlation coefficients as percentages. Basemap data in **a** from Natural Earth (https://www.naturalearthdata.com). Credit: icons in **b**, Freepik.com. BC, blue carbon management and marine revegetation; F, afforestation, reforestation and improved forest management; Fa, agroforestry; Fp, bio-based products; Fs, silviculture; Ft, timber in construction; RV, terrestrial revegetation and urban greening; SCS, soil carbon sequestration; and tW, terrestrial wetlands restoration.

Concerning in-boundary carbon removals, all 103 Mission Cities (Fig. 2a) intend to implement land-based temporary carbon sinks, with carbon sequestration being either the prime intention or a co-benefit (Methods). The most common practice is urban revegetation and greening (98% of the cities), followed by soil carbon sequestration (84%) and forms of forestation or improved forest management (80%), sometimes combined with silvicultural practices (23%) and/or agroforestry (19%) (Fig. 2b). The use of forested material to enhance or protract the carbon sequestration is scarcely intended by Mission Cities, with timber in construction and other bio-based products mentioned by only 16% and 13% of them. Terrestrial wetlands restoration and blue carbon practices (including marine wetlands restoration) are considered by 28% and 33% of the cities, encompassing all coastal cities. Commonly, Mission Cities combine three or four temporary CDR options (52%). Best practices include land planning provisions to protect local green from urbanization pressures, plans for carbon buffers as reserves within sequestration projects (Dunkirk and Zagreb) and provisions to maintain the health and ecosystem services of urban greening (for example, 20 cities refer to irrigation or similar; Supplementary Information). Some cities pay attention to tree and plant species (Mannheim, Zaragoza, Lahti, Paris and Glasgow) that are also heat- and drought-resistant (Warsaw), prioritize tree diversity (Barcelona), optimize prelogging forest growth period (Oslo) or use smart equipment to monitor and counteract reversals (Trikala and Dunkirk).

Permanent CDR is less prevalent among Mission Cities, with only 32% mentioning it, through BECCS (27%), biochar (13%) and DACCS (7%). Another 25% mention CCS without specifying the origin of the carbon (fossil or atmospheric). Only two cities (Helsinki and Helsingborg) include all three options (BECCS, DACCS and biochar), ten cities consider a two-option combination, while 21 cities include only one option. The most common combination of permanent CDR options is BECCS and biochar (seven cities). BECCS plants are typically on waste facilities (waste-to-energy plants and waste incineration), cogeneration or geothermal plants with hybrid biomass and fossil supply. Interestingly, the cities that plan to use BECCS or DACCS are located in countries with above-average underground carbon storage potential (CO_2_ storage indicator (CSI index) of 59.5 > 75th percentile; Methods). Further, 82% of the cities pursuing BECCS or DACCS are located in countries where the same solutions form also part of the national long-term strategies for climate change mitigation submitted to the United Nations Framework Convention on Climate Change (UNFCCC) under the Paris Agreement^39^. Permanent CDR is more popular among Nordic cities, whereas Southern Europe cities tend to fully rely on temporary CDR, with a few exceptions, for example, in Italy and Greece (Fig. 2a). The use of permanent CDR is more distinctive of cities with medium-high residual emissions per capita (33–35%). Among best practices (Supplementary Information), the cities of Stockholm, Malmö and Helsingborg emerge as pioneers of permanent CDR methods. For Stockholm, the large-scale BECCS facility linked to a biofuel-fired combined heat and power plant is anticipated to sequester more than the residual emissions of the city. Malmö and Helsingborg are also exploring BECCS linked to both waste-to-energy and district heating. Malmö is partnering with the main biochar innovation and development project in Sweden to use biochar in various municipal applications. Helsingborg is implementing a BECCS facility at its cogeneration plant, while also producing biochar, with a competence centre for biochar established to expand production and encourage its use in agriculture.

The third compensation option is carbon credits, included by 40% of the cities, of which only 39% provide information on the origin of the credits (for example, type of credit-generating projects and framework). Frequently cities explicitly mention the lack of credibility of currently available carbon credit markets as the main reason for excluding this option from their current compensation strategy. Resorting to carbon credits is characteristic of cities with high residual emissions (49%).

In total, 14 cities consider all three compensation options (temporary CDR, permanent CDR and carbon credits), 19 combine temporary and permanent CDR, 27 combine temporary CDR and carbon credits and 42 resort uniquely to temporary CDR. When DACCS or biochar are mentioned, forestation and/or revegetation tend to be excluded (Fig. 2c). Similarly, where timber in construction is part of the strategy, also other bio-based products are mentioned. Other strong positive correlations (>25%) exist between BECCS–biochar, DACCS–BECCS and among certain temporary CDR options (for example, agroforestry–timber in construction and forestation–soil carbon sequestration) (Fig. 2c).

## Strategic planning for robust residual emissions compensation

The residual emissions strategies are tested against emissions compensation options (ECO) and another five dimensions: residual emissions quantification (REQ), compensation potential (CP), timing (*T*), monitoring and verification (MRV) and overall governance (*G*), summarized by the residual emissions strategy robustness index (RESRI ∈ [0,1]; Table 1 and Methods).

**Table 1:** Dimensions and algorithm used to compute RESRI

Dimension	Objects of assessment	Rating
Residual emissions quantification (REQ)	Analysis (of origin of residual emissions and constraints in emissions reduction) versus target setting, clarity, completeness, internal consistency and baseline selection (historical, recent or business-as-usual—BAU—projection)	Five-point scale, where^a^: 4 = residual emissions quantification based on analysis of hard-to-abate emissions, clear and unambiguous magnitude, complete account of contributing sectors, recent or BAU baseline; 0 = no quantification at all.
Emissions compensation options (ECO)	Natural carbon sinks, technological carbon sinks and carbon credits. Special attention is given to distinguishing between emissions reduction/avoidance measures, which enhance the overall mitigation target (for example, fossil carbon CCS) and residual emissions compensation, which addresses emissions that still reach the atmosphere (for example, BECCS and DACCS).	Five-point scale, where^a^: 4 = compelling account of various locally applicable strategies (even within the same type of compensation measure, for example natural carbon sinks); no conflation of emissions reduction and compensation; 0 = no emissions compensation options (either no strategy or all measures are emissions reduction measures).
Compensation potential (CP)	Quantification (existence and accuracy) of emissions compensation potential across all, some or none of the considered options.	Five-point scale, where^a^: 4 = full and credible quantification of all options considered; 0 = no quantification.
Timing (*T*)	Inclusion of timelines and compatibility with the Mission Cities’ pledge to achieve climate neutrality by 2030.	Three-point scale, where^a^: 2 = timeline specified and compensation potential expected by 2030; 0 = no timeline specified.
Monitoring, reporting and verification (MRV)	Provisions for data collection and elaboration, monitoring, reporting and verification specifically for (1) residual emissions, (2) emissions compensation measures (outputs, outcomes or impacts including reversals and credits verification) and (3) total carbon sequestration.	Three-point scale, where^a^: 2 = MRV provisions in place, beyond intentions; 0 = no MRV provisions in place.
Governance (*G*)	Governance provisions that enable CDR and carbon credits deployment (for example, community-led initiatives, zoning and other regulation and incentives) or protect and legitimize CDR (for example, carbon buffers, precautions against double counting, maintenance, land preservation against urbanization and training in sustainable agricultural practices).	Three-point scale, where: 2 = precautions are taken to preserve sinks and account for their effect correctly AND actions are included to stimulate social acceptance, awareness and participation; 1 = if the AND is replaced with an OR; 0 = residual emissions strategies that leave all the above uncovered.

The scoring approach considers that the current residual emissions strategies are likely to be refined and improved in future CNAP iterations, as explicitly stated in many plans.

^a^Intermediate rates rely on evidence-based expert judgement.

$${\mathrm{RESRI}}=\frac{\sum ({\mathrm{REQ}},{\mathrm{ECO}},\,{\mathrm{CP}},T,{\mathrm{MRV}},\,G)}{\mathop{\sum}\nolimits_{i}\,\mathop{max}\limits_{\mathrm{score}}(i)\,{\mathrm{for}}\,i\,{\mathrm{in}}\,({\mathrm{REQ}},{\mathrm{ECO}},{\mathrm{CP}},T,{\mathrm{MRV}},G)}=\,\frac{\sum ({\mathrm{REQ}},{\mathrm{ECO}},\,{\mathrm{CP}},T,{\mathrm{MRV}},\,G)}{18}$$

**Fig. 3:**
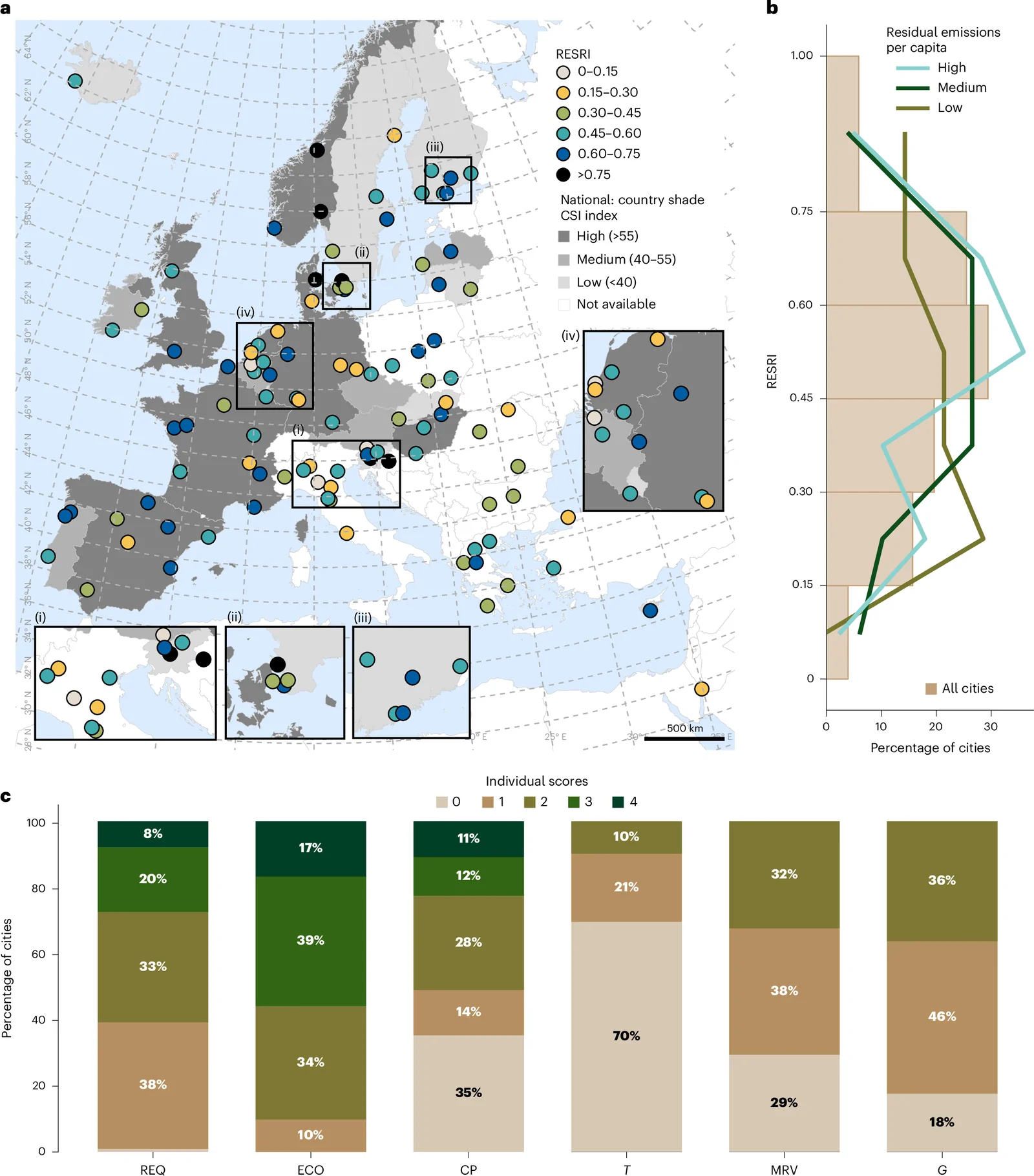
RESRI distribution and composition. **a**, Geographical distribution of RESRI values in 0.15-spaced bins overlaid on country shading based on the corresponding CSI index). Insets (i)–(iv) enlarge dense regions of the figure to enhance its clarity. **b**, Percentage of cities per RESRI bins for the entire ensemble of cities (rectangles) and for different levels of residual emissions per capita (polygon histogram). **c**, Percentage of cities across different RESRI components and scores. Basemap data in **a** from Natural Earth (https://www.naturalearthdata.com).

The median RESRI is 0.5, sitting right between very fragile and highly promising strategies. The distribution is skewed towards lower values (IQR 0.35–0.61), which makes less robust strategies more common. RESRI falls in the highest bin (>0.75) only in Nordic and Slovenian cities (Fig. 3a). However, the occurrence of RESRI in the 0.60–0.75 range appears geographically distributed. Italian and Eastern Europe cities are dominantly associated with the lowest bins. No statistically significant correlation was found with city size, gross domestic product (GDP) per capita or total green cover (as sum of forests, wetlands, coastlands and other vegetation) with Spearman rank correlation coefficients at 0.80, 0.77 and 0.20, respectively (RESRI is not normally distributed). Interestingly, RESRI commonly exceeds 0.75 in cities with low residual emissions per capita suggesting that cities that already invested in deep decarbonization have also advanced strategic planning for the compensation of any residuals (Fig. 3b). In this first action plans iteration, Mission Cities placed attention to comprehensive solutions mapping (ECO) and enabling conditions (*G*), while facing challenges in relation to quantification (REQ and CP) and performance tracking (*T* and MRV) (Fig. 3c and Box 1).

Box 1 City performance according to RESRI subindexes Only 8% and 11% of the cities reached the highest score (4) under REQ and CP, respectively, indicating that only a small subset of cities successfully quantified both residual emissions and the carbon compensation potential of their strategy (Fig. 3c). Two-thirds of the cities assessed the carbon sequestration potential of at least one option; yet only one-fifth were able to quantify compensation options that covered all their residual emissions. The share increases to half if cities mentioning carbon credits in their strategies intend to purchase sufficient credits to cover any remaining gaps (not explicit). Aggregated quantified compensations amount to 11.3 MtCO_2_ or about 18% of total residual emissions.

In other RESRI dimensions, Mission Cities performed substantially better. In total, 17% scored highest in terms of comprehensiveness of compensation solutions (ECO), with 46% scoring either 3 or 4 and none scoring 0. This entails that cities could define a variety of local compensation options and integrate them in their planning. However, these efforts are not always explicitly aimed at carbon sequestration, particularly in the case of nature-based solutions. Moreover, 31% of cities (*n* = 32) confused emissions reduction beyond 2030 and compensation measures, which may lead to inaccurate accounting of emissions reductions, targets and compensation efforts. It also suggests that by 2030, several Mission Cities would have not saturated the opportunity space for decarbonization.

Mission Cities almost never specified the timing within which the intended compensation would materialize (*T* = 0 for 70% of them) and lightly touched on monitoring provisions to track the evolution of local carbon sequestration (MRV = 0, 29%). Only 32 Mission Cities included key performance indicators dedicated to carbon sequestration within the CNAP monitoring framework or mentioned them. Nevertheless, a variety of best practices in monitoring and data collection emerge from the analysis (Supplementary Information) making use of ‘unsealing registries’ and vegetation cadasters (for example, Mannheim and Ljubljana), precision satellite technology and digital twins (for example, Zaragoza and Leuven), sensor networks and real-time monitoring systems (for example, Dublin), environmental digital models (for example, Glasgow, Riga and Grenoble-Alpes Métropole), comprehensive inventory and assessment systems (for example, Warsaw and Wroclaw) and citizen science and AI-powered methodologies (for example, Leuven and Dublin).

Governance (*G*) appears comparatively more robust, with Mission Cities using a range of institutional, regulatory, planning, financial and participative instruments to enable carbon sequestration (36% scored highest). Best practices include green infrastructure guidelines and standards (for example, Oslo, Helsinki, Riga and Zagreb), dedicated grants (Warsaw and Budapest) and public–private partnerships (Porto), community engagement initiatives (Barcelona, Porto, Trikala and Lahti) and public awareness campaigns (Leuven, Helsinki and Ljubljana). Cities such as Helsinki, Trondheim, Lisbon, Lodz, Aarhus and Angers Loire Métropole are conducting thorough analyses to inform their decision-making and show a cautious approach, calling for guarantees on potential, measurability, permanence, technological maturity and cost-effectiveness (Supplementary Information).

## Discussion, conclusions and recommendations

The 103 Mission Cities we investigated aim to reduce per capita emissions to 0.8 tCO_2_e/capita corresponding to a ninefold decrease from the EU27 average (6.9 tCO_2_e/capita in 2023; ref. ^37^) which suggests a deep decarbonization process^35^. This leaves 61 MtCO_2_e unaddressed by 2030 (around 20% of baseline emissions). The dominance of energy and transport emissions (50% and 31% of median residual emissions)—typically regarded as easy-to-abate sectors—raises concerns about the effectiveness of current strategies, the effect of imposing very strict net-zero timelines (2030) and the impact of typical urban constraints. These include limited influence over capital markets^40^ and restricted powers over certain sectors and stakeholders^6^, as also evidenced by the positive correlation between residual emissions and presence of (air)ports. Residual emissions might also be inflated by limited attention to demand-side measures that could decrease peak CDR use by 5–21% (ref. ^41^) and reduce emissions by 40–80% in end-use sectors^42^. Finally, the lack of an analytical approach to residual emissions, justified by vague notions of necessity (of certain activities and associated emissions to occur) and possibility (to decarbonize versus CDR)^8^ introduces a notable risk that cities may prioritize cost- or time-effectiveness over the transformative changes necessary for sustained emissions reduction, resulting in misguided investment and perverse outcome for climate action^43^. Most climate change mitigation scenarios are also cost-optimizing^44^ and not designed to keep CDR deployment within sustainability boundaries^45^ although this is rapidly evolving.

Higher-than-necessary residual emissions can lead to CDR over-reliance, a lack of accountability and a non-responsible stewardship of CDR resources. The Mission timeline may stimulate over-reliance, but it could also lay the groundwork for carbon-negative urban systems beyond 2030, as further emission reductions may remain achievable once neutrality is met. To avoid the downsides of CDR over-reliance, efforts can focus on high-co-benefit CDR options and on multilevel collaborative governance structures that bring together several stakeholders^46^.

Problematically, removal capacity falls short of what cities, countries and companies would need to deliver on their net-zero commitments^44,47^. National CDR pledges often reveal an over-reliance on land-use-related measures^48^ creating threats to biodiversity, food security, land rights and distributional justice^45,49,50^. Similarly, Mission Cities overwhelmingly rely on land-based CDR (42 exclusively) apparently without conducting a thorough assessment of land availability vis-à-vis urbanization, food, health or energy needs. The median Mission City is covered by forests at only 9% (IQR 2–27%), followed by other vegetation (21%, IQR 11–33%), wetlands and coastlands (1%, IQR 0.3–3%), whereas nearly 65% of the cities exhibit land-use efficiency <1 (IQR −0.3–1,4, median 0.5) suggesting sprawling dynamics. Hence, land competition might be a crucial bottleneck in the delivery of climate neutrality, if not explicitly considered and managed through integrated urban and territorial planning across governance scales. Further, using scarce land resources to compensate for emissions that are generally considered easy-to-abate can deplete reserves needed to hedge for Earth system feedbacks^51^, ultimately increasing system-wide spending^44^ and rendering net-zero goals achievable to a select few^43^. Finally, net-zero pathways may implicitly outsource carbon removal to rural hinterlands, raising risks of spatial inequity and political backlash.

Therefore, for cities, storing carbon in the built environment via bio-based materials—such as timber, hemp, straw and biochar as an additive to construction materials—takes on a strategic role as it capitalizes on existing and planned urban fabric to compensate for residual emission. A five-story mass timber building can store around 180 kg m^−2^ of carbon, about three times the carbon density of a natural forest with high biomass^52–54^. Provided that the harvested trees are replanted—sustainably restoring the forest biomass to previous levels—a substantial net increase in stored carbon could be achieved^55^. However, only one-sixth of Mission Cities consider this option. Biochar could similarly increase carbon storage in the built environment if used as a filler material in cementitious composites, with the potential to store 0.2–0.6 GtCO_2_ annually worldwide^32,33^. Recognizing and accounting for carbon storage in buildings can also catalyse a shift to sustainable materials, address often-overlooked scope 3 emissions and open new financing opportunities. The little attention paid by Mission Cities highlights a crucial missed opportunity for cities to transform their own urban fabric into a distributed carbon sink^52^. A notable exception is the city of Differdange in Luxembourg, which plans to integrate CO_2_ storage in concrete and wood into municipal tender dossiers to promote carbon storage.

The overarching scientific and political question is if, when and how cities may complement temporary CDR with more permanent options. In this first iteration, only 32% of the CNAPs mention BECCS, DACCS or biochar (predominantly BECCS on waste facilities). The co-location of national infrastructure and action planning for geological storage and the proclivity of cities to include BECCS/DACCS in their climate-neutrality plans, suggests the key role of national traction. As residual emissions intensity increases, permanent CDR becomes more necessary, with 68% of cities with medium-high residual emissions per capita considering this option, compared with 17% of cities with low residual emissions per capita. To address this need, national governments and stakeholders should collaborate to provide regulatory support, incentives and guidelines for permanent CDR. Finally, carbon credits are considered in 40% of the plans; however, (1) their use is not clearly defined, with the vast majority of cities lacking transparency on their plans for credit procurement, (2) they are typically seen as a last resort and (3) they are frequently questioned reflecting a widespread sense of distrust.

Our RESRI indicator shows that Mission Cities frequently laid a robust basis in terms of compensation options and governance architecture but faced challenges in avoiding double counting, verifying carbon sink potential and defining the timing for the intended compensation to materialize. This oversight is particularly concerning given the prevalence of tree planting and reforestation strategies, which may not yield notable carbon sequestration benefits until after 2030, potentially undermining the ability of cities to meet their 2030 climate-neutrality targets. In addition, the very limited quantification of CDR potential makes it difficult to assess the effectiveness of compensation strategies. To minimize the risk of undercommitment, cities may need to engage in more conservative estimations and robust calculations of both direct (carbon sequestration) and indirect (emissions reduction) impacts associated with, for instance, greening and urban heat island mitigation.

Overall, our analysis points to the need for a standardized framework for defining residual emissions^6^ and reporting transparently on their compensation. The European Commission released the first city-focused guidance in early 2026^56^ and harmonized certification frameworks for carbon credits are now emerging^57^. Efforts are required to translate these frameworks into practical tools for cities and better multilevel governance that front-loads emissions reduction, avoids entrenching inequities in burden-sharing and coordinates CDR portfolios according to local needs and broader sustainability goals^5,58^. Further, CDR governance frameworks should ensure the longevity of land-use and land-management decisions to protect carbon stocks from reversal^20^, in light of growing anthropogenic and natural pressures from urbanization processes and climate change^59,60^. Separate targets for emissions reduction and emissions compensation would decouple strategic planning in either direction from concerns of greenwashing, mitigation deterrence and moral licensing^43,61^. Consistent measurements and transparent reporting would also distinguish between genuine CDR enhancement and artificial improvements due to methodological changes^62^.

**Fig. 4:**
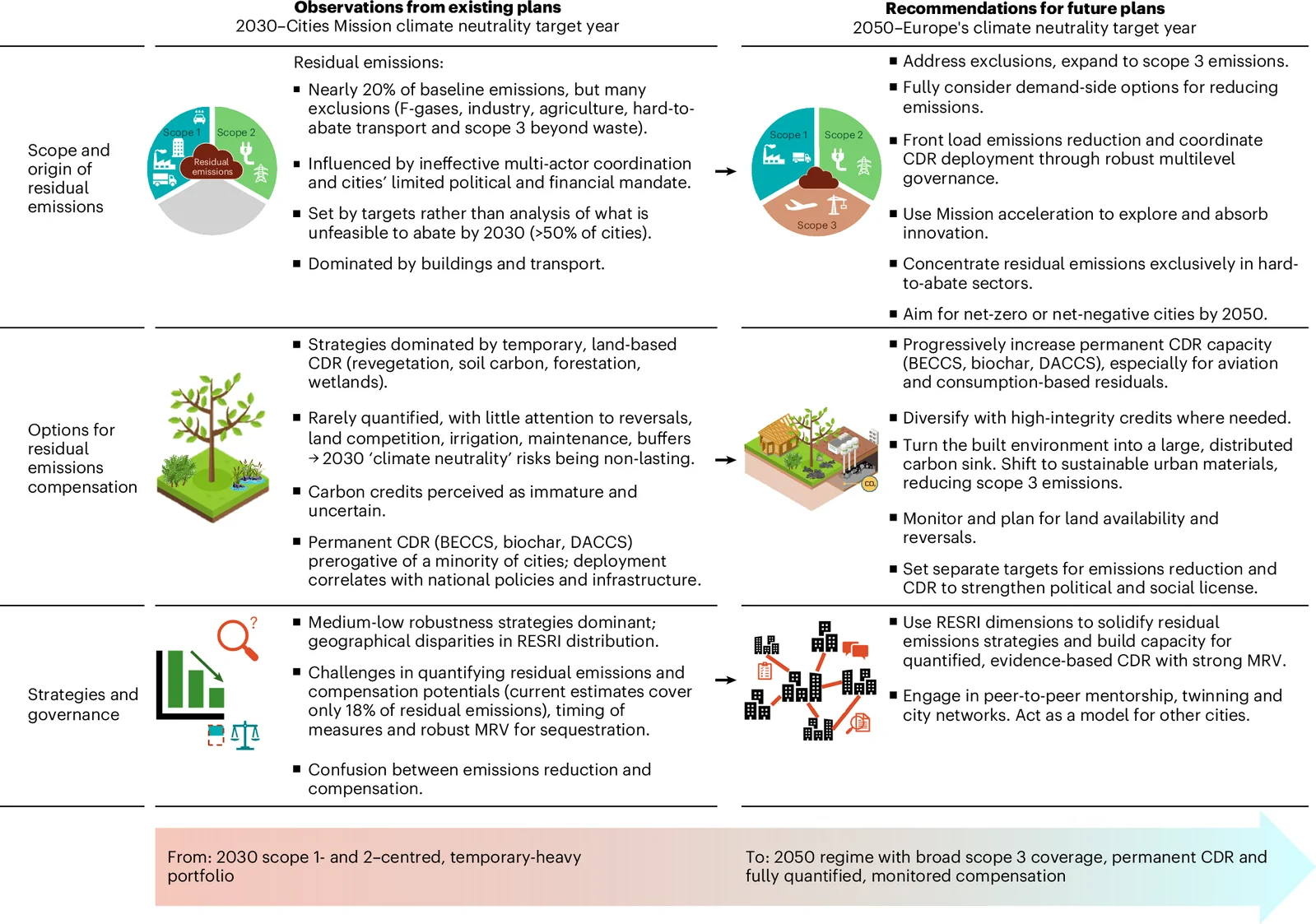
Learning from initial plans of cities for climate neutrality by 2030 to inform a climate-neutral urban Europe by 2050. Summary of key messages emerging from the analysis and relevant to the 2030 timeline of Mission Cities juxtaposed to the ensuing recommendations towards 2050. Credit: icons, Freepik.com.

Robust planning for residual emissions will be a crucial lever for ambitious local governments to demonstrate their climate leadership. As early adopters, Mission Cities could play a central role in shaping the policy and scientific discourse around CDR^6^. This analysis reflects their first attempts to collate preliminary planning on residual emissions and their compensation, clearly revealing substantial scope to improve the understanding and operationalization of urban CDR. The lessons that follow can guide a more targeted climate‑neutral trajectory for all urban Europe by 2050 (Fig. 4). Moreover, the mission-oriented approach initiated a process of deeper reflection and strategy development around emissions compensation. With CNAPs undergoing an iterative process every 2 years, regular stocktaking will disclose evolutionary patterns.

## Methods

The Horizon Europe Mission on Climate-Neutral and Smart Cities aims to deliver 100 climate-neutral European cities by 2030 and enable all European cities to become climate neutral by 2050. In the first stage of implementation, the Cities Mission succeeded in mobilizing a large cohort of cities across the European Union (EU) and associated countries (or countries in the process of negotiating association) to Horizon Europe to participate in an open call for expressions of interest (EOI). A total of 362 applications entered the EOI evaluation phase. On 28 April 2022, the Commission announced the 100 EU cities selected to participate in the Cities Mission. The 100 cities come from all 27 member states, with 12 additional cities coming from associated countries. The selected cities (Mission Cities) moved on to the next phase, that is, the development of climate city contracts (CCCs). The CCCs consist of three main parts: the main commitments, the CNAP and the climate-neutrality investment plan.

The template used to produce CNAPs contains a section dedicated to residual emissions strategies which offers the chance of a reality check. The Cities Mission operates on a specific definition of climate neutrality, which influences the magnitude of residual emissions presented in this Analysis. The definition foresees that by 2030 a net-zero balance will be established on emissions:

- from five accounting sectors: stationary energy (buildings, facilities, equipment, including public lighting), transport, waste (solid waste and wastewater), IPPU and AFOLU;
- covering fully scope 1 (direct in-boundary) and scope 2 (indirect in-boundary) emissions along with scope 3 (out-of-boundary) emissions associated with waste treatment and disposal;
- considering seven GHG: carbon dioxide (CO_2_), methane (CH_4_), nitrous oxide (N_2_O) and four F-gases, namely hydrofluorocarbons (HFCs), perfluorocarbons (PFCs), sulfur hexafluoride (SF6) and nitrogen trifluoride (NF3).

Beyond the Mission-aligned exclusion of most scope 3 emissions, Mission Cities could propose specific (justified) exclusions from the 2030 timeline. Hence, the net-zero targets outlined in the CNAPs are subject to additional exclusions, as illustrated in Fig. 1b. A deep manual analysis was conducted on each document to verify not only explicit exclusions, but also de-facto exclusions emerging from the lack of any activity, emissions data and dedicated action (logical AND), evident sectoral misallocations or city declarations implying additional exclusions. The magnitude of excluded emissions is not provided in the CNAPs; hence, a recalculation of total residual emissions is not possible. However, the volume of residual emissions associated with the 2030 climate-neutrality target is probably underestimated relative to the full scope of urban emissions, even considering only scope 1 and 2 emissions. Furthermore, CNAPs present emissions data in a fragmented manner, often with internal inconsistencies. In such cases, the residual emissions reported in this analysis reflect a sense-making process conducted by a team of experts. Commonly, residual emissions are established on the basis of a table contained in the CNAPs that juxtaposes baseline emissions, total emissions reduction by 2030 and the corresponding residuals.

In the framework of the Cities Mission, residual emissions are expected to stay below a ceiling of 20% of a recent baseline^38^. This ceiling is an orientation to stimulate emissions reduction at source as much as possible; however, cities are encouraged to determine the actual percentage of residual emissions analytically and not through target setting, in alignment with recent scientific direction^6^. This means that where the unavoidability of emissions is duly proved and discussed, residual emissions may exceed 20%. Equally, where achieving an emissions reduction beyond 80% by 2030 proves to be feasible, both target and overall planning should reflect this ambition. Cities are also invited to repeat the analysis and discussion of residual emissions at each CNAP iteration (expected every 2 years) to reflect how the local and global context have changed in terms of emissions reduction options and constraints. This approach operationalizes the dynamic nature of residual emissions.

### Study population

All CNAPs used in this analysis are publicly available^63^ and are in English. They were submitted by a total of 103 cities (two of which are in a joint plan) in four consecutive windows of submission between 2023 and 2025. Nine Mission Cities do not feature in this analysis as they are expected to submit their plans by 2026. The 103 Mission Cities are those that received the European Commission Mission Label as of October 2025, with the Label standing as an acknowledgement of the successful completion of the process followed by cities to develop their CCCs.

Urban administrations are the unit of analysis as the Cities Mission demands commitment from a single coordinating entity having authority to make decisions and commitments for the entire concerned territory (which may also contain suburban and rural areas). The city sample encompasses cities of 17,000 inhabitants as well as large conurbations of up to 17.8 million inhabitants (median of 319,000 inhabitants), covering 30 countries. The greatest representation (more than five cities) is of Italian, French, Spanish, Swedish, German, Finnish and Greek cities, in this order. Numerous governance arrangements are included, which may manifest as different degrees of maturity and integration of CDR and carbon credits in urban climate action. The sample includes a good balance between coastal and inland cities (41% versus 59%) and a variety of climatic conditions (all five main Köppen–Geiger classes but tropical climates)^64^.

The approach taken by a city to residual emissions was assessed on the basis of a coding exercise designed by experts within and beyond the Joint Research Centre of the European Commission. A suite of guiding questions, answer options and instructions were introduced to reduce risks of coding divergence across different evaluators. The coding was reviewed by all members of the team and subsequently piloted using three plans from the first window of submission (ten plans in total, April 2023). The inherent complexities and nuances associated with contextualized interpretation, juxtaposed with the de facto lack of specific information in the CNAPs, necessitated the implementation of an iterative refinement protocol to ensure the reliability and relevance of the coding. This adaptive methodology involved a cyclical process of questions, answer options and instructions recalibration, wherein successive reformulations and reconfigurations were undertaken in between the four discrete submission windows. The process aimed at guaranteeing an interrater reliability (same information coded in the same form by different evaluators) of at least 80% (refs. ^65,66^).

Beyond selecting the most appropriate answers among the options provided for the coding, experts were asked to supplement their evaluations with qualitative feedback, through written commentaries on aspects of the assessment of particular interest or prone to ambiguity. This supplementary input was provided in two primary forms: first, as annotations appended to specific answer choices, offering contextual insights and clarifications; and second, as concise textual entries in a standardized template, designed to elicit expert judgements on critical dimensions of comprehensiveness, credibility, feasibility and internal consistency.

### CNAPs analysis and data processing

To generate comprehensive and comparable information, this analysis relies on a labour-intensive, comprehensive process of individually reviewing and coding information across the CNAPs (with varying length from less than 100 pages to over 1,000 pages) through a common data collection protocol. A team of 61 trained experts and data collectors was involved in the analysis of the CNAPs. Through content analysis (both conceptual and relational analysis), coders conducted a systematic manual scrutiny of the CNAPs (texts, images and symbolic matter) to gather evidence.

Emissions data were manipulated to harmonize various sectoral contributions into the five main accounting sectors of the Mission mentioned above, in line with international practice. A sixth category ‘other’ was introduced to solve instances where cities themselves labelled emissions as ‘other’ or where associating emissions to only one of the five sectors was error-prone. In the second case, ‘other’ emissions typically contain a mix of IPPU, AFOLU and energy-related emissions associated with industry and agriculture. Baseline emissions were corrected to account only for gross emissions, disregarding sinks under AFOLU. Residual emissions are presented in this analysis as tonnes of CO_2_ equivalent (tCO_2_e) on an annual basis.

The strategies to compensate for unabated emissions were coded on the basis of the definitions contained in Extended Data Table 1. In addition, it was recorded whether the cities considered carbon credits or made generic mentions of CCS or CCU (carbon capture and utilization) with no clear indication on the origin of the captured CO_2_ (fossil versus atmospheric).

The robustness of the residual emissions approach and strategy is measured against the six main macrodimensions illustrated in Table 1), whose selection was informed by key scientific literature critically questioning the legitimacy of net-zero targets^1,5,6^ and dedicated initiatives, such as the UN Race to Zero campaign^67^. Two scoring scales are applied:

1. REQ, ECO and CP (0–4 scale)—these dimensions evaluate the core analytical integrity of the climate-neutrality pathway of a city: (1) quantification of residual emissions, (2) identification of compensation options and (3) quantification and balancing of carbon sequestration relative to residual emissions. Together, they represent the primary decision-making loop that determines whether a net-zero target is internally consistent and technically feasible. These components are tested for both their quantitative/procedural rigour and internal consistency. Higher scores therefore indicate stronger evidentiary grounding and credibility of the climate-neutrality claim (that is, whether the accounting framework demonstrably balances).
2. *T*, MRV and *G* (0–2 scale)—these dimensions assess enabling conditions for implementation and verification, namely timing, monitoring and governance arrangements. They function as facilitating layers that operationalize the strategy defined by REQ, ECO and CP. These subindexes are evaluated primarily on the presence, clarity and institutionalization of procedural elements.

The different ranges serve three methodological purposes. First, they prevent procedural indicators from exerting disproportionate influence over the composite index relative to analytically substantive dimensions. Second, they reflect differences in measurement resolution: quantitative methodological elements can be assessed with finer discrimination than institutional or procedural attributes. Third, they align score variance with evidentiary depth, ensuring that dimensions supported by more detailed and comparable data can express greater differentiation across cases.

The RESRI is calculated assuming equal weighting of each dimension. RESRI ranges between 0 and 1, with 0 representing a highly undetailed and not credible approach to residual emissions and 1 attributed to advanced strategies. It is used in this analysis to provide a comprehensive understanding of city-level performance and elucidate geographical patterns.

The resulting dataset is publicly available^68^.

### Additional data sources

A number of general city attributes (for example, climatic class^64^, coastal versus inland city^69^, GDP per capita^70^, presence of ports, airports within the geographic boundary and in a 10-km radius^71^, land cover from the Corine dataset^72^ and land-use efficiency (LUE) from the Global Human Settlement Layer^73^) were retrieved via GIS coding on the cities administrative polygons. LUE is a measure of the population growth in relation to land consumption in the same time frame (2010–2020). The analysis also makes use of the data on the EOI in the Cities Mission, a comprehensive questionnaire of over 370 questions in nine sections designed to provide for a systematic assessment of the starting point, commitment and capacity to reach climate neutrality by 2030 of a city^74^.

Additional data on national climate strategies on CO_2_ geological storage were retrieved from a stocktake by ref. ^39^ looking into the presence and prevalence of different options for CCS and subsurface CDR in national decarbonization pathways worldwide (long-term low greenhouse gas emissions development strategies submitted to the UNFCCC before 1 January 2024). The authors codified whether such strategies include BECCS, DACCS, natural carbon sinks, fossil CCS (relevant to emissions reduction) or a mix of fossil CCS and CDR. Further, the dataset includes the CSI index as developed by the Global CCS Institute^75^. It is based on technical aspects including geology, maturity of storage assessments, track record of storage projects, site characterization and technical ability to store CO_2_. Not all the countries associated with the 90 Mission Cities are available (Romania, Greece, Türkiye, Italy, Israel, Poland and Bulgaria do not appear in the dataset), resulting in the exclusion of 29 cities in the sample.

City and country attributes were used for pattern finding and city clustering. Statistical tests are two-sided.

### Reporting summary

Further information on research design is available in the Nature Portfolio Reporting Summary linked to this article.

## Online content

Any methods, additional references, Nature Portfolio reporting summaries, source data, extended data, supplementary information, acknowledgements, peer review information; details of author contributions and competing interests; and statements of data and code availability are available at https://doi.org/10.1038/s41558-026-02691-0.

## Supplementary information

## Data Availability

The dataset that underpins the analysis of the emissions compensation strategies is publicly available in the repository of the Joint Research Centre of the European Commission^68^, as part of a data collection on the Cities Mission: https://data.jrc.ec.europa.eu/collection/id-00450.

## Extended data

**Extended Data Table 1:** List and classification of residual emissions compensation strategies

Durability	Strategy (category & implementation options)	Definitions
Temporary CDR	Afforestation, reforestation and improved forest management **(F)** Specific implementation options: - Agroforestry **(Fa)** - Silviculture **(Fs)** - Timber in construction **(Ft)** - Bio-based products **(Fp)**	**(F)** Afforestation refers to the planting of new forests on lands historically dedicated to other use, or the restoration of the tree cover on minimally-covered lands, while reforestation refers to forests replanting on lands that contained forests in the past but were converted to other use; improved forest management focuses on increasing the resilience of existing forest land by changing the management practices, that is, without land-use change or demand for land conversion. **(Fa)** Agroforestry is the intentional integration of trees and shrubs into crop and animal farming systems to create environmental, economic, and social benefits. It comprises alley cropping, silvipasture, riparian forest buffers, windbreaks, and forest farming. **(Fs)** Silviculture is a practice to increase the forest carbon stock. It refers to the growing and cultivation of trees to control the composition, structure, and dynamic of a given forest. Silvicultural practices include interventions such as site preparation, selection of forest species and genotypes, managing forest nutrients and residues, undergrowth control, prescribed fires, and targeted tree thinning. **(Ft)** Timber in construction refers to using forestry materials and engineered wood products (such as glued laminated timber, glulam), oriented strand lumber, cross-laminated timber, engineered bamboo) or their recycled version in the construction sector to provide storage for the CO2 captured in forests. **(Fp**) Bio-based products refer to using primary (and secondary) biomass including from organic consumer waste, agricultural harvest and forestry residues to provide storage for the CO2 captured in forests.
	Terrestrial revegetation and urban greening (**RV**)	(**RV**) Common revegetation measures relate to increasing woody biomass outside forests, for example, in perennial crops, hedgerows, small islands of forest, shrublands, and urban and periurban greening (for example, tree planting, green belts and strips, urban parks, vegetated streets, ecological corridors)
	Soil carbon sequestration **(SCS)**	**(SCS)** SCS consists in changing land management practices to increase the soil carbon content. For croplands, options include i) crop management (that is, improved varieties and their rotation, use of ‘cover crops’, perennial cropping systems, and agricultural biotechnology), ii) nutrient management (that is, optimized fertilizer type, application rate, timing, precision application), iii) reduced tillage intensity and residue retention, and iv) improved water management (including drainage of waterlogged mineral soils). For grasslands, options include i) vegetation management (improved grass varieties, deep rooting grasses, increased productivity, and nutrient management), ii) animal management (stocking density, improved grazing management, improved animal feed production), and iii) fire management.
	Terrestrial wetlands restoration (**tW**)	(**tW**) It includes restoration of terrestrial wetlands like peatlands (that is, a type of freshwater wetland with an accumulation of partly decomposed plant matter) and constructed wetlands for wastewater treatment. It is typically obtained by rewetting the ecosystems by blocking drainage, among accompanying measures.
	Blue carbon management and marine revegetation **(BC)**	**(BC)** Management of rooted vegetation, including tidal marshes, mangroves, seagrasses (for example, Posidonia), salt marshes, coral reefs, ocean sediments, phytoplankton and macroalgae such as kelp. It can be pursued via marine revegetation and rewetting.
Permanent CDR	Bioenergy with carbon capture and storage (**BECCS** or BioCCS)	(**BECCS**) It involves capturing and permanently storing CO2 from processes where biomass is converted into fuels or directly burned to generate energy. BECCS plants may use purpose-grown biomass crops, cropping and forestry residues, and urban and industrial organic waste.
	Direct air carbon capture and storage (**DACCS**)	(**DACCS**) It consists in removing CO2 from the atmosphere by placing large volumes of air in contact with chemicals (sorbents). Sorbents are treated so that the CO2 is released from them, for sequestration in geological storage sites or for durable utilisation.
	**Biochar**	**Biochar** is a stable and long-lived charcoal-like product, produced by pyrolyzing (terrestrial or marine, primary or secondary) biomass in an anaerobic environment at temperatures ranging between 300–800 °C. In the process, the original biomass carbon is stored in a form that is relatively resistant to decomposition and that can stabilize organic matter added to the soil.

The table contains no references.

## Code availability

No code was generated or used to create or analyse the data.
