# Supplementary material for: Empirical insights into residual emissions in urban net-zero targets
Source: Nat Clim Chang. 2026 Aug 14;16(9):1092–101. doi: 10.1038/s41558-026-02691-0 (PMC13538053; doi:10.1038/s41558-026-02691-0)
Supplement: Supplementary file 1 [file 41558_2026_2691_MOESM1_ESM.pdf]

nature climate change

Supplementary information https://doi.org/10.1038/s41558-026-02691-0

Empirical insights into residual emissions in urban net-zero targets

In the format provided by the authors and unedited

Contents

BEST PRACTICES 3
Belgium (1 Best Practice / 2 Mission Cities) 4
Leuven 4
Bulgaria (1 Best Practice / 2 Mission Cities) 4
Sofia 4
Croatia (1 Best Practice / 1 Mission City) 5
Zagreb 5
Denmark (1 Best Practice / 3 Mission Cities) 6
Aarhus 6
Finland (2 Best Practices / 6 Mission Cities) 6
Helsinki 6
Lahti 7
France (5 Best Practices / 9 Mission Cities) 7
Paris 7
Dunkirk 8
Angers Loire Metropole 9
Nantes Metropole 10
Grenoble-Alpes Metropole 10
Germany (2 Best Practices / 7 Mission Cities) 11
Mannheim 11
Muenster 11
Greece (2 Best Practices / 6 Mission Cities) 12
Athens 12
Trikala 12
Hungary (1 Best Practice / 3 Mission Cities) 13
Budapest 13
Ireland (1 Best Practice / 2 Mission Cities) 14
Dublin 14
Italy (1 Best Practice / 9 Mission Cities) 14
Milan 14
Latvia (1 Best Practice / 2 Mission Cities) 14
Riga 14
Luxembourg (1 Best Practice / 1 Mission City) 15
Differdange 15

Netherlands (1 Best Practice / 5 Mission Cities) ... 16
Amsterdam ... 16
Norway (3 Best Practices / 3 Mission Cities) ... 17
Oslo ... 17
Trondheim ... 18
Stavanger ... 18
Poland (4 Best Practices / 5 Mission Cities) ... 19
Warsaw ... 19
Wrocław ... 20
Lodz ... 21
Rzeszow ... 22
Portugal (2 Best Practices / 3 Mission Cities) ... 22
Lisbon ... 22
Porto ... 23
Slovenia (2 Best Practices / 3 Mission Cities) ... 23
Ljubljana ... 23
Kranj ... 24
Spain (2 Best Practices / 7 Mission Cities) ... 24
Barcelona ... 24
Zaragoza ... 24
Sweden (3 Best Practices / 7 Mission Cities) ... 25
Stockholm ... 25
Helsingborg ... 25
Malmö ... 26
Türkiye (1 Best Practice / 2 Mission Cities) ... 26
Izmir ... 26
United Kingdom (1 Best Practice / 2 Mission Cities) ... 27
Glasgow ... 27

BEST PRACTICES

The following section presents a curated collection of best practices that supplement the article's findings. The identification of best practices is based on a multi-faceted evaluation, independent of a city's RESRI score, and considers various aspects (including integrated urban planning, going beyond carbon sequestration) and individual components of the RESRI framework. The geographical distribution is illustrated in **Figure S1**. For clarity, the descriptions are organised by country, with best practices from the same country grouped together and presented in alphabetical order by country name.

Out of 30 countries including all EU27 Member States, no best practices were found in Austria, Cyprus, Czechia, Iceland, Israel (each represented by only one Mission City), Lithuania, Slovak Republic (two Mission Cities), or Romania (three Mission Cities). In addition, some countries showed low prevalence of best practices, notably Italy (1 best practice / 9 Mission Cities), Germany (2/7), Spain (2/7), and The Netherlands (1/5). In contrast, other countries demonstrated a variety of best practices, including Norway (3/3 cities), Poland (4/5 cities), Portugal (2/3 cities), Slovenia (2/3 cities), and France (5/9)

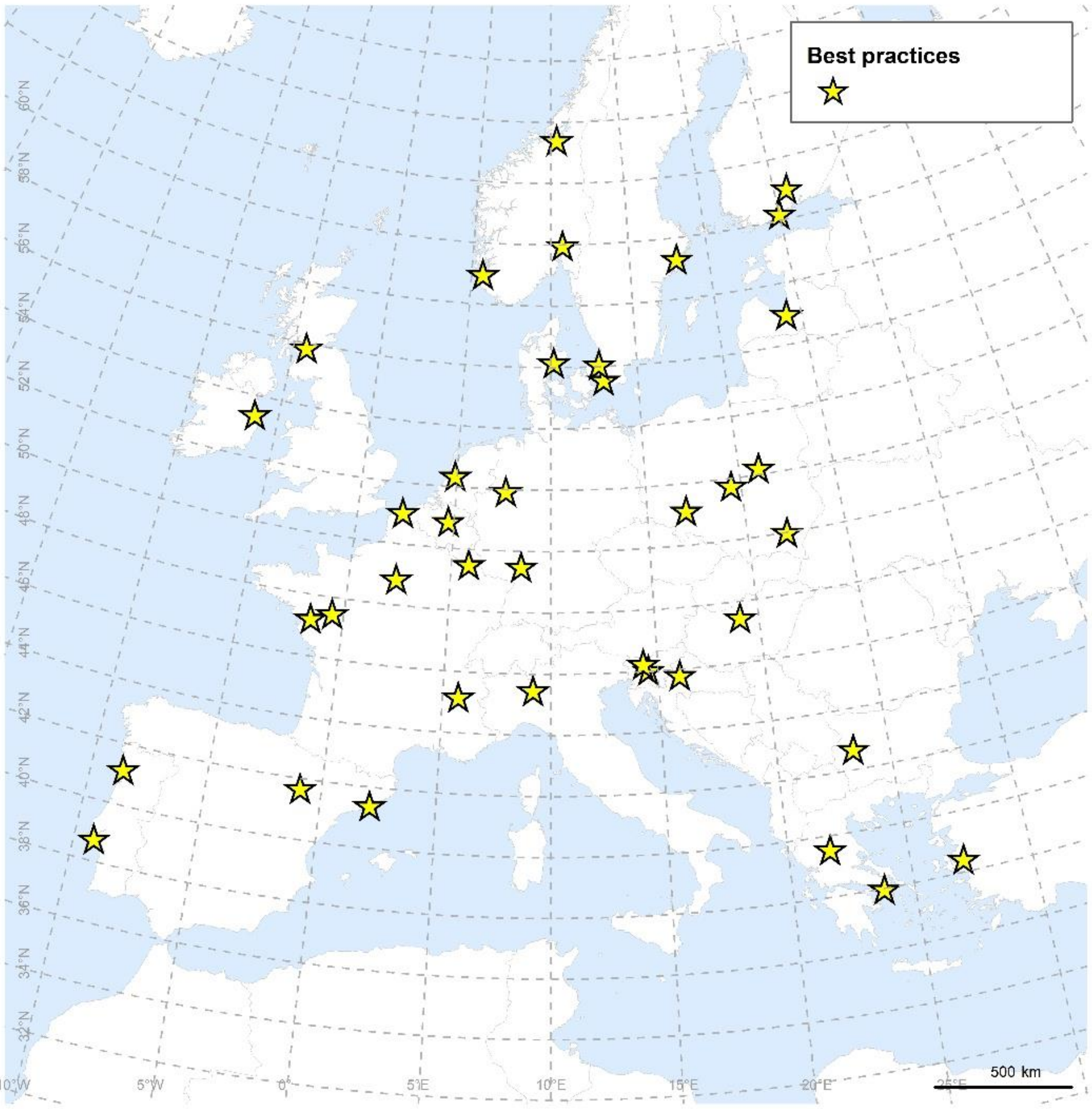

Figure S1. Map of best practices.

Belgium (1 Best Practice / 2 Mission Cities)

Leuven

Leuven proposes a comprehensive approach to carbon sequestration in close connection to climate change adaptation goals. The city's measures focus on protecting and reinforcing existing carbon sinks and implementing green infrastructure, carbon farming, and nature-inclusive farming projects. To monitor the effectiveness of these interventions, Leuven utilises a robust monitoring system, including a digital twin, citizen science projects, and a comprehensive set of key performance indicators such as net increase in the number of trees, in the area of "neighborhood forests" and in the number of green walls, surface area of agricultural land where carbon sequestration measures have been implemented, percentage of buildings where the 3-30-300 rule is met, and structural connectivity and accessibility of green spaces. The city's governance structure promotes public awareness and involvement through campaigns like "Let It Sink In" and encourages citizen engagement through a climate adaptation toolkit.

Bulgaria (1 Best Practice / 2 Mission Cities)

Sofia

The city of Sofia has implemented a comprehensive approach to natural carbon sinks. The city's measures include the rehabilitation and expansion of existing parks, gardens, and green areas, as well as the planting of new trees and creation of a green belt around the city. Additionally, Sofia is developing new forests, implementing soil management practices to improve topsoil and carbon sequestration, and restoring areas as habitats for various species. Sofia's governance structure plays a crucial role in supporting these efforts, with a strong emphasis on participatory models and stakeholder engagement. The city has implemented initiatives such as the EU-funded CITIES4CSR project, which has helped to prioritise areas of action, including greenery and green infrastructure, and has engaged various stakeholders, including branch organisations, NGOs, and universities. The city has also empowered young people to take climate action through projects like Food Wave and has established a culture of citizen consultation and participation through events and projects like ECO PAVILIONS and Sofia Chooses. The city has also developed a plan to support the long-term watering of tree vegetation and other green areas in conditions of prolonged summer and autumn droughts. This plan aims to ensure that trees and shrubs receive adequate water during critical phases of their development in unfavorable climatic conditions. Moreover, each year, old trees are replaced by new more appropriate species as per the scientific recommendations, new trees are planted in the city and a new forest is developed to surround the city and protect it from outside harmful emissions impact. Furthermore, Sofia has initiated actions to develop expert catalogues of trees and other plant species suitable for urban landscaping and agricultural crops and varieties suitable for use in agriculture given the expected future climatic conditions. Sofia has also implemented innovative measures such as the creation of "blue-green" arches for coolness in urban environments and blue and green acupuncture. These initiatives involve developing and implementing small-scale solutions to enhance urban adaptation to climate change, focusing

on creating integrated spaces with water and greenery in public areas prone to heat islands. The city is also implementing landscape measures to accelerate tree growth, including improving soil composition, using compost and natural fertilisers, and optimising the structure of tree massifs and accompanying vegetation.

Croatia (1 Best Practice / 1 Mission City)

Zagreb

The City of Zagreb presents a clear strategy for compensating residual emissions by 2030. The city's approach includes a range of measures such as green belts, agroforestry zones, and peri-urban corridors, green roofs, permeable pavements, retention basins, wetland restoration, timber in construction and biobased materials, soil carbon management into zoning for peri-urban agriculture, carbon farming, desealing, planting native trees and low shrubs, landscaping lawns, rain gardens, and biodiverse flower meadows, preparing beds for growing vegetables and herbs, installing green pergolas, collecting rainwater for the maintenance of green spaces, and leveraging certified removals, such as carbon credits, if necessary.

The city's governance structure is well-established, with residuals explicitly embedded into the Green General Urban Development Plan, the Green Deal Building Design Standards, and the city's governance architecture. This ensures that carbon sinks protection and enhancement is spatially planned, technically standardised, and institutionally anchored. The city has also developed a range of initiatives and instruments to support its carbon sequestration efforts, including the ActGreen project, which aims to expand the green space system by greening private and semi-private areas, such as residential and commercial building courtyards. The city's Green Deal Design Guidelines go beyond national minimum standards, requiring mandatory climate proofing of all new projects, integration of on-site carbon sinks, circular construction and biobased materials, and Do No Significant Harm (DNSH) and EU taxonomy alignment. The city's Climate Cockpit governs residuals, with cross-departmental ownership and public transparency, ensuring that residuals and removals are tracked on a public dashboard alongside emissions reductions.

The city's financing strategy for residuals is also well-established, with mainstream investment, including European Investment Bank (EIB) framework loans, Cohesion Policy 2021–2027 and Recover and Resilience Facility (RRF) funds, and private capital and ESCO models, mobilised for bio-based construction and materials banks. The city also facilitates the uptake of Common Agricultural Policy (CAP) eco-schemes and carbon farming pilots by farmers and cooperatives. The city's approach to carbon sequestration delivers a range of social and economic co-benefits, including jobs, resilience, equity, and urban quality. The city's stakeholder engagement process involves collaborating with industry representatives, agricultural stakeholders, and forestry management entities to co-create actionable strategies and ensure accurate data inputs.

The city's monitoring efforts are comprehensive, with green belts, agroforestry zones, and peri-urban corridors tracked as verified carbon sinks, and residuals integrated into the city's monitoring, reporting, and verification (MRV) system. The city proposes annual residual emissions inventories by sector, MRV protocols for removals, and public transparency, with

residuals and removals tracked on a public dashboard alongside emissions reductions. The city also monitors the area of new green spaces, the number of new trees planted, the number of new shrubs planted, new natural retention areas established, and rain gardens implemented.

Denmark (1 Best Practice / 3 Mission Cities)

Aarhus

Aarhus strategy is based on an extremely rich analysis of the status quo, which leads to a comprehensive approach that encompasses various initiatives to sequester carbon and promote sustainable land use. Its key strength lies in its holistic planning, integrating multiple initiatives such as reforestation, set-aside of lowland soils, and biogenic energy parks, to create a cohesive plan for compensating for emissions. With ambitious targets, collaborative partnerships, and innovative solutions, the strategy optimises land use for multiple purposes, including recreation, biodiversity, and carbon sequestration. This is complemented by the "A Greener Aarhus" strategy, which reflects a Nature-Based Solutions (NBS) approach, prioritising the conservation and restoration of natural ecosystems to address environmental challenges. This approach is evident in the strategy's four focus areas: Nature in the open country, The green elements in the city, Experiences and stays in nature and green surroundings, and Co-creation on green projects, emphasising nature restoration, conservation, accessibility, and co-creation with citizens, associations, and other stakeholders. The monitoring setup involves a variety of output, outcome and financial indicators.

Finland (2 Best Practices / 6 Mission Cities)

Helsinki

Helsinki is approaching the compensation of residual emissions in a highly evidence-based manner. It has conducted three separate consultant analyses to inform its decision-making process, including calculating existing carbon sinks, exploring possibilities for increasing natural sinks and technical sinks, and examining compensation programmes, regulation, challenges, and costs. The city has also made cost-impact analyses of all possible means, including various compensation mechanisms, technological sinks, and additional actions in emission reduction. This approach is driven by the need to address uncertainties in regulation, financial viability, and political/social acceptance, including the challenges of verifying potentials and avoiding double counting. In parallel, Helsinki has incorporated adaptation measures into various programmes and developed tools, such as the stormwater programme, green roof guidelines, and the green factor tool. the city has updated its building regulations to include a lot-specific green area factor (which is now mandatory when applying for a construction permit, ensuring that new developments include sufficient green infrastructure). Within the ARVO project, partially funded by the EU, the city aims to expand the green area factor from individual lots to larger public areas. Furthermore, Helsinki is preparing for increased rainfall by assessing potential rain scenarios and developing a hydrological model, which will lead to large-scale green infrastructure projects. The city is also evaluating tree canopy cover across districts to establish minimum coverage levels, further strengthening green

infrastructure. The city is leveraging innovative tools, such as the "Carbon Negative Helsinki" board game, to raise awareness and understanding of a carbon-negative future, probing and stimulating public acceptance around compensation of residual emissions. The city is also participating in projects that study and promote the use of biochar and is networking with other operators to stay up-to-date on the latest developments in this field.

Lahti

Lahti's measures focus on reforesting underutilised areas, enhancing protected areas, utilising city-owned rental agricultural land for sequestration, and promoting tree-diverse projects. Lahti also aims to increase the use of carbon-storing materials in construction, such as wood, and promotes the production and purchase of local carbon credits. The city's governance structure is robust, with a dedicated sector for carbon sinks and offsetting in its Climate Programme, which outlines 19 actions for 2023-2030 to address residual emissions. Lahti's MRV system is noteworthy for its transparency, using net-carbon sink calculation models and a carbon map to track progress. The city is also developing a local compensation model and is participating in various projects and initiatives to gather knowledge and expertise.

France (5 Best Practices / 9 Mission Cities)

Paris

Paris is preserving and enhancing its green spaces, including creating a "trees and climate laboratory" and sanctuarising 100,000 street-lining trees and 250 remarkable trees. The city's management plan maintains an ideal level of organic matter in its soils and considers carbon criteria in urban project decision-making. The Coopérative Carbone Paris et Métropole du Grand Paris supports and finances regional offsetting projects, including agroforestry projects in the Paris Basin. The city promotes sustainable food systems, including urban agriculture and community gardens, which reduces its carbon footprint and enhances biodiversity. Paris is also participating in relevant EU-funded projects, such as the Urban Innovative Actions, LIFE, and Horizon Europe programmes. These initiatives bring together citizens, businesses, and local authorities to co-create solutions. The city's approach is characterised by a strong emphasis on collaboration, innovation, and community engagement. The "Paris Action Climat Biodiversité" network and the Climate Academy are examples of innovative governance models that bring together stakeholders to address climate and biodiversity issues. The city's efforts to preserve agricultural land and support sustainable crops also contribute to reducing emissions from land use change. Additionally, the city's foresight tool helps to consider carbon sequestration potential in urban planning decisions. The city's green spaces are also being enhanced through the "Green up the hearts of blocks and building courtyards" initiative, which opens up green spaces to the public. The city also pays attention to species selection, using Cerema's Sésame tool to choose tree species that have a high carbon storage capacity, ensuring that its urban forestry efforts are optimised for carbon sequestration.

Dunkirk

Dunkirk's approach to carbon sequestration features as a best practice, as it i) encompasses almost the full range of measures except for DACCS, ii) demonstrates unique attention to carbon reversals, and iii) enshrines a deep attention to biodiversity, along with a robust governance and monitoring structure. The city's initiatives include reforestation, improved forest management, restoring natural watercourses, tree planting, wetlands and watercourses restoration. Additionally, Dunkirk is promoting urban carbon farming, soil enhancement, and the use of carbon-negative materials, such as carbon-absorbing concrete or aggregates, and biobased technologies that utilise carbon-storing materials like hempcrete, recycled plastic, or other biomaterials. The city is also focusing on agroforestry, silviculture, rewilding and urban ecosystem restoration. Temporary sinks are complemented by small-scale BECCS plants integrated with local waste management systems, biochar in urban agriculture and landscaping, and carbon credits.

The strategy is supported by a robust governance framework, which includes a multi-faceted approach to mitigation, monitoring, and contingency planning and makes use of sustainable land use, urban planning, and community engagement. The city has implemented a biodiversity coefficient to encourage the construction of green roofs, and is working with companies like Ecocem and ECOPAL to promote industrial and regional ecology. The local authority's commitment to biodiversity is evident in its collaboration with the local biodiversity office, and its efforts to create a green and blue network aligned with pedestrian and cycling needs. The city has also launched initiatives to promote sustainable agriculture, reduce food waste, and support urban agriculture, such as the Territorial Food Project and the development of a food hall. Furthermore, Dunkirk is engaging citizens in sector-related initiatives through participatory approaches, such as the Youth Assembly, and educative programs, like the Climat Libé Tour. The city's governance framework also stands out for the attention on reversals. To minimise the risks associated with non-durable emissions sequestration, Greater Dunkirk has developed a comprehensive approach that combines:

- Monitoring and early detection: establishing long-term monitoring systems to regularly assess the stability and effectiveness of natural carbon sinks, enabling early detection of potential reversals.

- Buffer reserves: creating buffer reserves within carbon sequestration projects to ensure a portion of carbon offsets or captured carbon remains untapped as a safeguard against unexpected reversals.

- Insurance and risk management: investing in insurance mechanisms and carbon risk management funds to address financial losses and ensure continued compliance with the climate neutrality target if reversals occur.

- Durability enhancement: prioritising the integration of carbon capture technologies into infrastructure projects to store carbon permanently. By implementing this multi-faceted strategy, Greater Dunkirk aims to strengthen its overall approach to achieving long-term climate neutrality.

Dunkirk's monitoring protocol is designed to track the effectiveness of its carbon sequestration efforts and identify areas for improvement. The city is using a range of indicators, including the number of species present on site, area of preserved natural areas, and biodiversity index. Additionally, Dunkirk is tracking the share of organic farming, agro-ecological practices, and certified forest area, as well as the number of trees planted and forest sequestration. The monitoring protocol also includes the use of geographic information system (GIS) visualisation to spatially represent environmental data and identify areas for improvement.

Angers Loire Metropole

Angers Loire Métropole, with the city of Angers, also known as the "capital of plants", has a comprehensive approach to carbon sequestration, which co-targets biodiversity conservation, urban cooling, air quality, and ecosystem services. The metropole's measures to achieve carbon sequestration include an increase in forest biomass, changes in farming practices to increase carbon storage in soils and hedgerows, such as cover cropping techniques, increase in hedgerow planting, and a doubling of the current rate of conversion to agroforestry. Additionally, the metropole is implementing measures to increase urban woodland, tree planting, changes in land use and timber harvesting in the forest, desealing, renaturation, and desilting, as well as promoting organic farming, parks, and gardens, and despoiling schoolyards.

The metropole 's governance structure is well-established, with a range of initiatives and instruments in place to support its carbon sequestration efforts. The metropole is committed to gradually reducing the rate at which land is artificially developed, with the aim of achieving zero net artificialisation by 2050, and is incorporating the zero net artificialisation (ZAN) objectives into urban planning documents. The metropole is also implementing the ZAN objectives in new development projects, including large-scale mixed projects, housing, business parks, and urban renewal projects. Furthermore, the metropole has set an objective to triple carbon stocks by 2050 and is strengthening its biodiversity plan, including discovering, raising awareness, and training to improve collective action, sharing and pooling to improve knowledge, preserving and restoring to protect the natural heritage, innovating and experimenting to meet new challenges. The metropole is also implementing the Angevins landscape master plan and the Urban Nature Plan, which aims to strengthen the area's landscape identity, sustainable design and management, and promote the tree heritage.

The metropole 's approach to carbon sequestration is also focused on promoting sustainable agriculture and food systems, through the Territorial Food Project, which strengthens local food systems, promotes organic agriculture, and addresses social accessibility. It is also engaging in partnerships, agroforestry transitions, and community-led projects, such as urban gardens, to promote cross-sectoral alignment. The governance approach is characterised by a participatory approach, with stakeholder collaboration and localised action, as evidenced by the Ecological Transition Conference and citizens' roadmap. The metropole is also leveraging digital tools, such as sensorisation for real-time water monitoring, and exploring digital twin technologies for smarter resource management.

The metropole 's monitoring efforts are comprehensive, with a range of key performance indicators (KPIs) tracked to assess progress towards its carbon sequestration goals. These include the annual artificial surface area, annual surface area desilted, number of trees planted, net sequestration of carbon dioxide from soils and forests, change in surface area of forests and undeveloped areas, and proportion of agricultural area certified as organic or in conversion and of high environmental value. It also includes relevant indicators to track co-benefits, such as air quality, or number of people affected by heat island effects. Each action to achieve climate neutrality has at least one monitoring/results indicator, and regular reporting of this data is carried out through numerous strategic documents and action plans, as well as annual reports of the Urban Community's public services.

Nantes Metropole

Nantes Metropole proposes a comprehensive set of measures to expand natural carbon sinks, such as restoration of rivers, marshes, and ditches, urban forestry, agroforestry, bio-sourced materials, and the creation of "Urban Freshness Islands" to provide cooling effects and improve thermal comfort. The metropole also aims to implement the 3-30-300 rule, which states that every inhabitant should be able to see 3 trees from their home, benefit from at least 30% tree cover, and have access to a tree-covered "Îlot de fraîcheur" no more than 300 meters away.

The metropole 's governance structure is well-established, with a range of initiatives and instruments in place to support its carbon sequestration efforts. It has created a structure to store carbon and increase ecological resilience, which includes local financing of natural carbon storage and environmental restoration projects, aggregation of private funding, and provision of consultancy services to help territories assess their existing carbon stocks and potentials. The metropole also has a Metropolitan Tree Charter, which was validated in 2024, and has implemented a range of actions to restore the region's rivers, marshes, and streams to a good ecological state. The metropole's approach is characterised by a strong emphasis on climate change adaptation, biodiversity conservation, and sustainable land use practices. It has implemented a range of measures to reduce the consumption of agricultural, natural, and forest spaces, and has established a Metropolitan Observatory to monitor the consumption of these spaces. The metropole also has a range of initiatives to promote ecological resilience, including the creation of a carbon and ecological resilience engineering and investment company, and the development of a financing system to support local projects.

The metropole 's monitoring efforts are focused on tracking the increase in CO2eq capture in the territory, the consumption of agricultural, natural, and forest spaces, and the status of water bodies. The metropole also monitors the number of km of rivers restored, the number of Urban Freshness Islands, and the status of water bodies.

Grenoble-Alpes Metropole

Grenoble-Alpes Metropole's measures include maintaining and strengthening carbon sequestration in forests, wetlands, and agricultural soils, increasing urban canopy cover, depaving, and promoting agroforestry. Additionally, the metropole is encouraging the use of timber in construction, implementing BECCS at its incinerator, and exploring carbon capture

solutions and local recovery of the captured CO2 in district heating. The metropole is also considering the use of carbon credits to offset its emissions.

The inter-territorial forestry strategy, launched in 2019, aims to encourage local use of wood and develop carbon sequestration, while the Chartreuse forest and wood label promotes sustainable management of the resource, the environment, and carbon sequestration. The Regional Wetland Restoration Plan and the Agricultural Strategy also include actions to maintain and strengthen carbon sequestration in wetlands and soils. The Canopy Plan, launched in 2022, aims to increase the number of trees in urban areas and contribute to carbon sequestration, with a goal of reaching 30% canopy cover by 2030 and 40% by 2050. The use of timber in construction is encouraged through the decarbonisation objectives for new buildings set out in the Land Use Regulation Plan. Furthermore, the metropole 's commitment to collaborative climate action is evident through social innovation actions, like the Citizens' Convention for the Climate, and its participation in EU-funded projects, such as Food Trails, CityZen, and Climaborough.

In terms of monitoring, the metropole carries out an annual GHG/Energy/Air assessment on a territorial scale, as well as monitoring of observation indicators, all brought together in the Climate Observatory Letter. Relevant KPIs include the net annual rate of change in carbon stocks per hectare of land, the amount of permanent sequestration of GHG within the metropole boundary, and negative emissions through natural sinks.

Germany (2 Best Practices / 7 Mission Cities)

Mannheim

Mannheim is focusing on climate neutrality in conjunction with climate adaptation, biodiversity, and improved quality of life, placing a strong emphasis on enhanced urban forests and better forest management. In planning afforestation, Mannheim is making use of recent and innovative concepts behind carbon sequestration and climate adaptation (e.g., in the use and mix of biotypes). In addition, the city is creating an "unsealing register" to keep track of the level of permeability associated with land use, coupled with a tree cadastre. These measures would facilitate integrated urban planning and reduced uncertainty in estimations. The overall intention is to implement the concept of the sponge city in a sustainable way, with positive repercussions in terms of carbon sequestration and beyond. Green areas and the unsealing of settlement and traffic areas contributes to reducing urban overheating and improving or restoring the buffer function of the soil during rainfall events.

Muenster

Muenster's carbon sequestration strategy prioritises af- and re-forestation, moorland rewetting, biochar, and restoration of green infrastructure to minimise emissions. The city's approach to governance emphasises stakeholder and citizen involvement, raising awareness through education, and collaboration with regional policies. The city's Agricultural Officer plays a key role in fostering communication with local farmers and integrating agricultural perspectives into climate protection projects. A comprehensive nine-month soil practitioner course, jointly

organised with agricultural associations, educates farmers on carbon storage, soil protection, and water retention capacity. The city's educational initiatives, such as Climate walks, aim to raise awareness and stimulate action among citizens, while also promoting synergies with regional policies.

Greece (2 Best Practices / 6 Mission Cities)

Athens

The city of Athens discusses a very compelling governance of natural carbon sinks, with strong emphasis on collaboration with national and local agencies, academic and research institutions, and private entities. The city has established an extensive plan for land expropriation, upgrading of hills and groves, and reinforcement and restoration of streams, and has set ambitious goals for tree planting, with a target of planting over 35,000 trees by 2030. The city is also increasing funding sources for green infrastructure, hiring new staff, and providing training on nature-based solutions for municipality departments, citizens, and other stakeholders. The city's approach to governance is also characterized by a strong focus on social equality, with initiatives such as the GreenInCities project, which aims to address urban inequality by improving the microclimate, sustainable mobility, and accessible infrastructure for all ages and social groups.

In terms of monitoring, Athens has made significant progress in 2024, with the establishment of a geodatabase to record and monitor tree plantings and the launch of the AthensTrees application, which allows citizens to monitor tree plantings in real-time. The city has also established a new regulation on green infrastructure, strengthening its commitment to a sustainable and green future. Additionally, the city has implemented an Integrated Framework of Actions for the digital upgrade, development, and modernisation of the infrastructure and equipment of the Department of Greenery and Urban Wildlife.

The city's efforts are also supported by the "Cooling Havens" project, which was approved in 2024 and envisages water-based and nature-based projects. The project aims to improve the city's resilience to heatwaves and promote sustainable development.

Trikala

Trikala's approach to natural carbon sinks emphasises the importance of monitoring and governance around natural carbon sinks. Strategically placed small measuring stations will record the rate, degree, and manner of influencing urban green spaces, while IoT sensors and remotely controlled electrovalves will enables autonomous energy systems for irrigation, guaranteeing that natural ecosystems can stay healthy and sequester as much carbon as possible. The city's commitment to precision agriculture technology and smart farming algorithms allows for data-driven decision-making, enhancing the efficiency of urban farming initiatives. The creation of citizen teams for urban farming and green roof maintenance ensures community engagement and ownership, while also promoting local food production and reducing reliance on imports. Regular maintenance plans for green pavements and urban green

spaces are also recognised as crucial to ensuring their effectiveness and longevity. Finally, a variety of KPIs are in place to track the realisation of effective carbon sequestration.

Hungary (1 Best Practice / 3 Mission Cities)

Budapest

The Municipality of Budapest proposes a comprehensive carbon sequestration programme through urban greening and conservation efforts. These include enhancing and developing park infrastructures, regenerating floodplain forests and riparian habitats, greening of courtyards, roofs, and walls, preserving and expanding forest cover, planting and protecting trees in public spaces, renewing green spaces within residential areas, and developing green infrastructures within cemeteries, including the promotion of forest or tree burials. Additionally, the city is implementing measures such as reforestation and afforestation, greening of brownfields, enhancing existing green infrastructure, protection and sustainable management of existing forests, increasing forest areas, developing an outer green ring, renewal and replacement of public trees, new tree planting, greening courtyards, roofs and walls, wetland revitalisation, improving water connectivity and green corridors, revitalisation of natural assets of stream valleys and riverside, floodplain forest regeneration, rehabilitation, and habitats protection, and promoting the uptake of urban agriculture. Overall, the city is considering every greening option, using all suitable urban surfaces, from brownfields to roofs, from riverside and watercourses to cemeteries, to maximise its carbon sequestration efforts. The city is also open to collaborating with the private sector and research hubs on $CO2$ removal technologies, and encourages the use of carbon credits for private actors with limited capacity to eliminate emissions within the city border.

Several plans and initiatives are also in place. The Radó Dezső Plan and the Green Infrastructure and Maintenance Plan provide a framework for the city's integrated recreational and green space development. The Green Budapest Foundation aims to improve the environmental condition of the green capital, protect and maintain green spaces, and support local environmental and nature conservation initiatives. The city also offers grants and tenders to support civil and public initiatives that promote a greener city and has established community gardens to provide opportunities for residents to practice urban gardening and promote sustainable living. Furthermore, the city plans to develop or update regulations and plans to protect trees, including tree protection and compensation ordinances, and will establish a 2030 timeframe for afforestation with an annual timetable. The city's strategic development plans for urban parks, development of reforestation programmes, and regulation of public space use are also key components of its governance approach.

The city's approach stands out for the commitment to biodiversity, social and cultural functional value and heritage, and climate adaptation beyond carbon sequestration. This is evident in the various efforts to ensure the maintenance of forest and other semi-natural ecosystems and habitats, and to promote recreational development aligned with nature conservation aspects. The city's placemaking initiatives, which aim to renew and utilise urban spaces according to community needs, are a testament to its dedication to creating liveable and sustainable

environments. The establishment of the Department for Climate and Environmental Affairs in 2019 has been a key driver of these efforts, solidifying environmental protection as a key pillar of city governance. The city's participation in relevant EU-funded projects, such as LIFE projects and Horizon projects, demonstrates its commitment to addressing pressing issues such as flood adaptation, sustainable food systems, and habitat rehabilitation, while also reimagining its urban spaces to be more inclusive, green, and resilient.

Ireland (1 Best Practice / 2 Mission Cities)

Dublin

Dublin proposes advanced monitoring of local carbon sequestration through the Urban Sense pilot project, which has deployed 20 sensors across the city to measure greenhouse gases, air quality, and weather variables in real-time. This initiative is part of the broader Terrain AI research collaboration, which aims to develop innovative AI and computational methodologies to improve the measurement, monitoring, verification, and reporting of carbon stocks and emissions across complex environments. By leveraging these advanced monitoring technologies, Dublin is poised to gain a deeper understanding of its carbon fluxes and develop more effective climate mitigation strategies, ultimately supporting the delivery of targeted interventions to sequester carbon and address social and environmental challenges. The project's focus on providing accurate estimates of carbon fluxes and identifying cost-effective policies will enable the city to maximise co-benefits and demonstrate the potential of climate action initiatives through digital twinning.

Italy (1 Best Practice / 9 Mission Cities)

Milan

Milan cherishes the social dimension around activities linked to carbon sequestration. The "Metropoli Agricole" annual conference brings together stakeholders to discuss solutions and approaches towards sustainable agriculture. The city's initiative to facilitate collaboration and knowledge sharing among experts, policymakers, farmers, and researchers has helped address the lack of a unified understanding of climate-neutrality in agriculture. Furthermore, Milan's Cooperative Urban Gardens project has promoted social interaction, knowledge sharing, and environmental awareness among residents, while also enhancing community cohesion and social inclusion.

Latvia (1 Best Practice / 2 Mission Cities)

Riga

Riga's approach to carbon sequestration uses exclusively natural carbon sinks. It includes the establishment or restoration of blue-green spaces, such as urban meadows and waterfronts, trees and shrubs planting, green roofs and facades, urban agriculture and community gardening, permeable street surfaces and nature-based rainwater solutions, new forest stands and the management of existing stands, enhancement of net sink and reclamation of peat bog areas, and the creation of ecological networks.

The development of a Street Greenery Renovation Plan and a Riga Urban Environment Greening Plan 2027-2031, as part of the EU-funded project LIFE LATESTadapt, aims to enhance the city's green infrastructure and promote sustainable urban development. The city is also developing a Biodiversity Strategy, which will further support the creation of an enabling environment for carbon sequestration. The city is also prioritising plants in the greening that have low resource demands and are resilient to the impact of climate change, and is installing beehives on the roofs of municipal buildings. Additionally, the city is developing a regulatory framework to increase CO2 sequestration and the implementation of nature-based solutions, including the revision of land use and development regulations, the development of a 'green factor' that takes greening and nature-based solutions into account, and the amendment of the Protection Zone Law to promote the greening of streets. The city is also providing information, education, awareness-raising, and engagement opportunities for local residents and businesses, including the development of guidelines for local resident participation in the greening of urban areas, the engagement of businesses in the greening of urban areas, and the provision of support mechanisms for local residents and businesses.

In terms of monitoring, the city is tracking a range of key performance indicators, including CO2 emissions accumulated by the forestry sector, changes in tree canopy cover or forest area, amount of green infrastructure by type, and total share of bodies of water. The city is also using remote surveying methods to map and monitor urban green spaces, and is developing and improving its data records system and emissions calculations. Additionally, the city is commissioning a data analytics project to plan the potential development of Riga and enable forecasts of theoretically deforested areas in Riga's forests in the future. The city is also analysing unused urban land to select additional areas that can be afforested to compensate for deforestation needed for infrastructure.

Luxembourg (1 Best Practice / 1 Mission City)

Differdange

Differdange's strategy for addressing residual emissions is comprehensive and foresees both nature-based solutions and carbon credits, combining local and international initiatives. Locally, it promotes urban greening (e.g., green facades, roofs, and agroforestry) and mandates carbon-capture construction materials (CO2-infused concrete, certified timber) via regulatory reforms. It also sells municipal carbon certificates and supports regional projects like habitat restoration and reforestation with ProSud/TNT. Internationally, Differdange collaborates on CCS/CCU projects in the Netherlands and Norway and purchases international offset certificates. Initiatives include promoting green public spaces, forest bathing, green roofs and facades, supporting local farmers, and exploring carbon capture and storage solutions. The city also plans to integrate CO2 storage in concrete and wood into municipal tender dossiers, and to create a municipal system for the sale of local CO2 storage certificates. Notably, the DiffPrimes project aims to increase the number of fruit trees in the city and promote green facades for 500 buildings, focusing on capturing greenhouse gases through tree growth and establishing a recognised supply chain for green products. The project involves various initiatives, including collaborations with local stakeholders, feasibility studies, and the use of

innovative materials like carbon-captured concrete and CCU wood, with the goal of increasing carbon compensation in construction and promoting sustainable development. The city is also considering the use of hybrid Paulownia trees for carbon capture and the development of a natural insulation production chain using local materials. The city's collaboration with the Luxembourg Institute of Science and Technology (LIST) and ArcelorMittal to explore CCS solutions also demonstrates its commitment to innovative and effective compensation measures. The promotion of forest bathing and the creation of "instagrammable" places in the forest are also creative approaches to raise public awareness of environmental issues related to urban planning and protect carbon sinks. The city operates in a favourable policy framework for protecting and enhancing carbon sinks, with a strong focus on urban planning, green spaces, and sustainable land use. The city's initiatives, such as green roofs and facades, urban forestry projects, and agroforestry, are supported by national frameworks like the "Naturpakt" and international certifications like FSC and PEFC. The strategy prioritises innovation, scalability, and collaboration with regional and international partners, underscoring the city’s commitment to amplifying its impact on global climate change. By integrating social acceptance and broader environmental benefits into its compensation strategies, such as promoting green public spaces, cultivating transparency in the selection and management of offset projects, and supporting local farmers, the city demonstrates a thoughtful and holistic approach to emissions compensation, one that balances environmental, social, and economic considerations.

Netherlands (1 Best Practice / 5 Mission Cities)

Amsterdam

Amsterdam’s residual emissions strategy include BECC(U)S on the waste plants, biochar from green waste, wooden or bio-based materials in construction, enhanced park-like public areas, new large green areas, tree planting, recreational lawns, depaving, nature-inclusive construction, nature-friendly banks and embankments, green roofs, urban forests, parks and community gardens, urban farming and agriculture, organic and regenerative agriculture practices, and restoring soil health through composting and circular agriculture. Additionally, the city is exploring the use of carbon credits, although these are currently not considered trustworthy.

The city's governance structure is well-established, with a range of initiatives and instruments in place to support its carbon sequestration efforts. The city is involved in the City CDR initiative and is developing a LIFE application oriented towards using waste from parks to create biochar and verifiable credits. The city is also investigating possibilities for financial contributions from residents or private parties, such as in the case of a “CO2 forest” programme, where residents and businesses can contribute to the planting of trees to offset their carbon emissions. The city's governance approach is underpinned by stakeholder collaboration, circular economy principles, and policies that mainstream nature-inclusive design in urban planning. The city applies a "nature-inclusive building" points system in tenders commissioned by the municipality, requiring proposers to demonstrate how they integrate nature-based solutions into designs. The city is also engaging residents, businesses, and organisations in greening initiatives, providing knowledge, and encouraging participation.

The city's approach to carbon sequestration is also focused on protecting and enhancing biodiversity, including creating wildlife-friendly habitats and reducing pollution. The city is incorporating nature-inclusive design and construction principles into new buildings and infrastructure projects, including using sustainable materials, reducing energy consumption, and promoting green roofs and walls. The city is committed to making its own roofs and courtyards greener, and every new building complies with the rainwater regulation, which requires the collection and treatment of rainwater.

Concerning monitoring, a range of performance metrics is proposed to track carbon sequestration goals, including having a park-like public area no more than 10 minutes' walk from any home in 2050, and having a large green area no more than 15 minutes' cycling from any home in 2050. The city is also monitoring the use of wooden or bio-based main load-bearing structures in new residential buildings, with the aim of having 20% of new buildings using these materials by 2025.

Norway (3 Best Practices / 3 Mission Cities)

Oslo

The strategy for addressing residual emissions in Oslo is very credible. The CCS facility at Klemetsrud, expected to start operating in the summer of 2027, will not only avoid fossil emissions from reaching the atmosphere but will also capture 170,000 tonnes of biogenic CO2 (BECCS) enabling Oslo to become carbon negative by 2030. Oslo will nevertheless continue working on reducing its emissions, demonstrating a commitment to addressing climate change from multiple angles. In addition to technological sinks, the city brings forward an exemplary portfolio of actions for natural sinks preservation and expansion, without expecting any contribution to residual emissions compensation. The City Council has approved 13 mitigation measures concerning the management of the city's forests. Furthermore, Oslo's land-use policies promote densification and multi-aged and mixed forests through selective harvesting and natural rejuvenation. The Agency for Planning and Building Services is revising the regulations concerning the blue-green factor, a tool used to safeguard blue-green structure in construction projects. The city is also revising the land-use section of the municipal master plan, laying foundations for Oslo to become a climate-resilient city. The "Oslo Trees" project, which aims to plant 100,000 more trees, represents an important contribution to this work. Further efforts will be carried out to increase the cycle period prior to logging, determining the overall logging level and thinning intensity based on climate considerations, assessing more climate-optimal utilisation of timber from thinning, and continuing and further developing the work on wetland restoration. The focus is on restoring eelgrass in the Oslo Fjord to improve biodiversity and carbon uptake, while preventing land degradation and non-indigenous species proliferation. Further, an assessment will be made as to how, through the stipulation of requirements in procurements, the city can grow demand for slow-growing and sustainable timber of larger dimensions, which in turn can contribute to durable, high-quality timber products, and with an increase in the proportion of timber which is utilised.

Trondheim

The city of Trondheim has emerged as a model for effective carbon sequestration, showcasing a comprehensive approach that integrates analysis, measures, governance, and monitoring provisions. Trondheim's strategy begins with the analysis of the origin of residual emissions, a clear distinction between fossil and biogenic CCS, and a critical approach to natural carbon sinks such as forests and wetlands concerning the time needed to achieve maturity and significant carbon sequestration.

Hence, the city acknowledges that by 2030, there won't be sufficient time to significantly strengthen natural carbon stores to compensate for residual emissions. Thus, while planning and implementing long-term measures to enhance natural carbon sinks, the city also focuses on a BECCS installation on the waste-to-energy plant, capable of exceeding the full compensation of residual emissions. Regardless, the plan channels a firm commitment to nature and farmland protection, restoration, and reinforcement against climate change, forestation, urban farming, peatlands restoration, and sustainable agriculture.

Trondheim's governance structure plays a pivotal role in supporting carbon sequestration efforts. The city has implemented area-neutral regulations, which ensure that any new development or land use does not come at the expense of natural carbon sinks. Additionally, the city has introduced incentives for landowners to restore and protect natural habitats, making the real cost of reducing nature and agricultural land visible in the early planning stages of projects. The city provides funding for nature restoration, both from the public and private sectors, and has established partnerships with NGOs and agricultural organisations to promote sustainable land use practices. The city also engages its citizens in the decision-making process, raising awareness about the importance of viable nature and farmlands through information campaigns and participation projects. For example, Trondheim has launched urban farming initiatives and citizen science mapping projects, which not only increase community involvement but also provide valuable data for monitoring and evaluating the city's carbon sequestration efforts. Furthermore, the city uses its public procurement power to promote local food production, supporting farmers who adopt sustainable practices and reducing the city's reliance on industrial agriculture.

Monitoring and evaluation are also crucial components of Trondheim's approach, involving the mapping of land use, assessment of carbon stores, and identification of areas with potential for nature restoration. A database is being developed to map carbon-rich nature areas, with a focus on peatlands and forests. Key Performance Indicators (KPIs) such as natural carbon stocks are used to track progress.

Stavanger

Stavanger's action plan has a focus on measures to enhance urban greenery, protect natural areas, and promote sustainable development. The city's measures include tree planting, soil desealing, better soil health in agricultural land, and preservation and development of carbon-rich marine nature such as seagrass meadows, kelp forests, and coastal wetlands. The city has implemented a guiding provision in its municipal master plan to include the principle of area

neutrality for natural values in future land use planning, which aims to balance the maintenance of biodiversity and nature's contribution to people's lives while allowing development and urbanisation to continue. The city has also set a goal to redistribute no more than 24 acres of agricultural land for other land-use objectives each year and to physically alter or urbanise no more than 66 acres of agricultural land per year. The city's Green plan outlines its approach to green infrastructure, natural diversity, and outdoor recreation, and includes provisions for the protection of natural areas and the restoration of developed areas to a more natural state. Stavanger has also implemented a programme to combat alien species, proactively works to improve its knowledge base about carbon-rich areas and important nature, and is conducting supplementary surveys and comprehensive surveys of urban trees and their ecological benefits. In terms of monitoring, Stavanger has developed a first-generation map of carbon-rich areas to estimate the effect of planned land use change and is updating the map with enhanced data to support the development of its next municipal master plan. The city is also mapping carbon-rich marine sediments and supplementing mapping of carbon-rich marine nature. Additionally, Stavanger has delegated management responsibility for 27 nature conservation areas and is working to protect several new areas. The city's efforts are also supported by the use of carbon credits and the implementation of a BECCS plant for waste incineration.

Poland (4 Best Practices / 5 Mission Cities)

Warsaw

Warsaw's strategy to carbon sequestration is characterised by a comprehensive approach to preserving the environment, protecting biodiversity, and promoting sustainable practices. The city's measures to increase natural CO2 sequestration include maintaining and developing urban forests, parks, and squares, as well as greening backyards, streets, and transport tracks, while creating rain gardens, retention basins, and desealing its surface. The city will select vegetation with increased resistance to heat and drought and will consider technological sinks and carbon credits should natural sinks be insufficient to compensate for residual emissions.

Warsaw has established a range of policies and programmes to support natural sinks. The Environmental Programme aims to protect the surface of the earth and water from pollution and degradation, while the Municipal Revitalisation Programme focuses on eliminating threats to the natural environment and building resilience to climate change risks. The city also has a number of community and citizen initiatives, such as the Million Trees for Warsaw programme, which encourages residents to suggest planting sites using an app. Furthermore, Warsaw has introduced regulations and incentives to support green initiatives, including the Warsaw Greenery Board, which cares for and finances the care and development of green spaces in the city, and the Green Fund, which provides financial support for green projects. The city's efforts to promote green infrastructure are further supported by its Municipal land buy-back programme, which allows the city to acquire land for green spaces. The Warsaw Climate Change Adaptation Guidebook provides education and support for residents, cooperatives, and businesses to remove concrete surfaces, plant greenery, and set up small-scale retention facilities. The guidebook is complemented by information and education activities, such as workshops and meetings for residents, to raise awareness about the importance of green

infrastructure and climate change adaptation. The city also promotes community involvement through initiatives such as the Warsaw Booster acceleration programme, Urban Living Lab, and Participatory Budgeting, which support the development of innovative solutions in the sector. To encourage private sector participation, Warsaw has introduced subsidies for removing concrete and retention, especially for residents, and promotes so-called "green projects" related to retention, removing concrete, and greening. The city also implements the Warsaw Green Building Standard, which supports the implementation of retention tasks by municipal entities and then the private sector. To support the development of green infrastructure, Warsaw has established a concrete removal grant system and cooperates with partners within the framework of the Partnership for Climate. The city also develops accessible green spaces close to where people live and engages residents and the private sector in monitoring and protecting nature-valuable areas through citizen science activities, provision of tools, and family allotments as biodiversity sites. Additionally, Warsaw aims to introduce a uniform reporting system for the entire city and is building a system of incentives for the private sector, including financial support for private investments. Finally, the city is introducing provisions in local spatial development plans to protect and develop green and blue infrastructure. The Municipal Adaptation Plan will also include a dedicated inventory, diagnosis of the state of blue-green infrastructure, and an assessment and monitoring system for ecosystem services. The city information collection system, the City Information Bank, will provide a comprehensive and standardised collection of qualitative data concerning climate protection and the environment.

Wrocław

The city proposes several measures to increase natural CO2 sequestration, including afforestation, creating a park in every neighborhood, implementing the 3-30-300 rule, and promoting green roofs and facades. Additionally, Wroclaw is rehabilitating areas of biodiversity value, enhancing existing parks and green spaces, and creating new ones, including pocket parks and urban farms. Wroclaw has established an ambitious Urban Greening Plan, which aims to protect and develop blue-green infrastructure, increase the area of green spaces, and promote sustainable development.

The city is implementing a range of greening programmes and initiatives, including the "Green Lungs of Wroclaw" and "Park on every estate", while the Stormwater Management Strategy and programmes such as "I like rain" and "Catch the rain" stimulate unsealing of concreted surfaces, retention of rainwater, and prevention of flooding. The city is also introducing provisions in local plans to protect and develop blue-green infrastructure, and providing funds in the city budget for the purchase of land with natural values to protect it from urban pressure.

The city is encouraging residents, cooperatives, housing communities, and entrepreneurs to deconstruct pavements, plant greenery, and set up small retention facilities on land owned by them, through education and support programs, including expert advice from municipal entities and grant programmes for small retention facilities. Innovative initiatives, such as the "GROWin WROclaw" programme, which involves planting trees with newborns and young children as patrons, the "Green Culture" municipal programme, or the "Green Patronage"

programme, which encourages residents and businesses to sponsor green solutions in the city, and the "WROchef" competition, which promotes a plant-based diet among elementary school children; it partners with restaurant chefs and finalists of MasterChef to promote the competition and provide prizes, such as cooking with the finalists, to encourage children to develop healthy and sustainable eating habits. Also, the "Wroclaw Magnolia" competition, which aims to promote innovative projects and solutions that have a positive impact on air quality and biodiversity, and the "Saturday with Green Wroclaw" family picnic, which introduces residents to the city's sustainability activities, are also notable examples of the city's commitment to engaging citizens and stakeholders in creative and inclusive ways. Wroclaw is also promoting community involvement and social innovation through initiatives such as the "Wroclaw Participatory Budget" and the "Civil Panel", which allow residents to contribute to the development of a sustainable and resilient urban environment.

Wroclaw's monitoring system is also well-developed, with several indicators to track progress in the deployment of green sinks and co-benefits, the percentage of the population living within 300m of green areas, the area of the basic green system with adaptive functions, and the share of high greenery (tree canopy cover) which would also serve the purpose of monitoring the mitigation of urban heat island effects. The city is also developing a spatial database, including smart solutions for the inventory of greenery and assessment of ecosystem services, biodiversity, and naturally valuable areas.

Lodz

The City of Lodz proposes a clear plan for carbon credits, which specifies the types of projects, their origin, and crediting frameworks along with an estimation of the share of residual emissions covered (93%) and of costs involved (EUR 7-14 million). The types of projects to be considered include carbon forests, sustainable farming practices, peatland habitat restoration projects inter alia with a focus on projects in Poland and other EU countries or EU neighbouring states. The attention to credits does not entail disregard of other options, but a critical analysis of their (limited) potential. Delivering on a multipurpose agenda, the city is targeting: revitalisation of parks and green areas, creation of new urban forests and parks, creation of pocket parks near residential buildings, greenery along the city's public transport communication system, restoration and revitalisation of river valley areas, and other urban greening activities. The city is also exploring the use of BECCS, in consultation with industry.

The city's governance structure is well-established, with a range of initiatives and instruments in place to support its carbon sequestration efforts. The city has developed a Greening Plan till 2030 with the perspective till 2050, which involves active participation of residents at the planning and implementation stages. The city is also engaging in participatory budget mechanisms, grant support, and creating an inventory of trees using a special website. The main goal of the Greening Plan is to increase the amount and functionality of greenery in the city to support the achievement of climate neutrality, improve the quality of life of its inhabitants, and increase the attractiveness of the city for current and future generations. The city's approach is complemented by various social innovation actions and participatory approaches, such as the Ekopakt initiative, which allows businesses and stakeholders to financially support projects

related to green infrastructure and climate change mitigation. The city has also established a Civic Budget, which enables residents to submit and vote on projects, including those related to green infrastructure and climate change mitigation. The city engages with residents through citizen panels, such as the 2nd Lodz Citizens' Panel, which discussed climate change risks and solutions.

Rzeszow

Rzeszow's strategy to carbon sequestration describes the use and development of agricultural areas, forests and wetlands, along with tree planting, green roofs and façades, urban green acupuncture, green corridors, unsealing of urban surfaces, and parks revitalisation. A robust governance framework is in place, which includes a broad stakeholder partnership, particularly with the private sector. The city has developed planning documents, such as the Study of Conditions and Directions for Spatial Development and the General Plan, which prioritise green areas and their sustainable development. Rzeszow's planning documents take into account the need to protect areas for the development of green spaces and establish minimum biologically active areas for plots or land. Rzeszow has also established a range of participatory mechanisms, including the Citizens' Budget, the Social Dialogue Commission for Tree Protection, and the Green Fund, which promote sustainable land use, urban planning, and community engagement and enable residents to participate in decision-making and support the deployment of green infrastructure projects. The city is also promoting environmental education and social support for actions related to maintaining urban areas and is shaping pro-ecological and civic attitudes among city residents. The city is using analytical tools, such as the Obliview platform, to support spatial planning and decision-making and is conducting a comprehensive inventory of natural resources, including canopy maps, tree species and condition, and orthophotomaps. Notably, the Interactive Tree Canopy Map will provide valuable insights into the development of urban greenery.

Portugal (2 Best Practices / 3 Mission Cities)

Lisbon

The residual emissions strategy is in its early stage, but it already considers all compensation options (temporary and permanent CDR and carbon credits). The focus is on natural carbon sinks (forest, trees, shrubs, grassland, green areas, estuary) with important points of reflection on other forms of compensation. Concerning technological sinks (CCS/CCU) and carbon credits, the city raises concerns on the need for guarantees (on effectiveness, measurability, additionality, permanence and absence of double counting), the technological maturity, and the cost-effectiveness. The city looks forward to the planned creation of the Portuguese Voluntary Carbon Market and is reflecting on possible roles to take (buyer, investor, developer). The city also flags the need to develop additional guidance from the perspective of cities. This critical thinking might be inspired or highlighted by the city's attention to monitoring its carbon sequestration rate.

Porto

The city of Porto has implemented a comprehensive strategy for carbon sequestration, focusing on enhancing natural sinks through a variety of measures like afforestation, park creation and organic farming as well as studying the carbon sequestration potential of its soils, trees, and water bodies. The city will engage in the Voluntary Carbon Market in Portugal, to promote out-boundary carbon sequestration projects while protecting biodiversity and natural capital. The city leverages a dedicated Municipal Greening Plan, public-private partnerships, and community engagement initiatives, such as the "If You Have a Garden, We Have a Tree for You" programme and the 100,000 Trees project. The city has also established incentives for sustainable practices, including reduced fees for operations that promote green spaces and environmental policy contributions. Additionally, Porto invests in sustainability education and citizen engagement, with initiatives like the "Horta à Porta" project, which promotes organic farming and community involvement. Effective monitoring is ensured through detailed mapping and ledger of urban trees, allowing for real-time management.

Slovenia (2 Best Practices / 3 Mission Cities)

Ljubljana

Ljubljana’s strategy stands out for breadth of solutions, exemplary governance, and effective monitoring. Solutions include expanding forests and improving forest management, creating green roofs and facades, surface unsealing, establishing new green areas and urban parks, tree planting, restoration of field boundaries and riparian zones on Ljubljana’s marshes, promoting timber construction, expanding agricultural land adapted to climate change, establishing green mobility corridors and green parking areas, agroecology, agroforestry, and regenerative farming to improve city fruit self-sufficiency, revitalisation and greening of courtyards and atriums, sustainable farming and agricultural practices, public orchards, and community gardens, green vertical ecosystems, rain gardens, and carbon capture and utilisation technologies. The city's governance structure is robust, with a clear plan and budget in place. The city has also established a Green Infrastructure and Nature-based Solutions department and developed guidelines for transitioning to climate-adapted agriculture. Incentives are in place to encourage sustainable practices, such as financial incentives for investments in agricultural efficiency and promotion of local vegetable production. The city monitors its progress through dedicated KPIs. Additionally, Ljubljana has established a vegetation cadastre and is expanding its water level monitoring systems. Ljubljana's strategies include developing a Long-term Urban Greening Plan to increase urban green spaces to 40%, preparing a green roof and facade strategy, introducing comprehensive river system management to balance human needs and ecosystem preservation, and formulating policy promoting sustainable organic farming, reducing unauthorised interventions and damage to forests, and effective supervision and monitoring of greening projects. The city is also proactive in its land policy, protecting agricultural and undeveloped land, preserving green wedges, prioritising land acquisition for the development and preservation of green spaces, promoting sustainable land use, and integrating climate objectives into spatial planning.

Kranj

The city of Kranj has demonstrated a robust approach to estimating residual emissions and calculating sequestration potential, acknowledging that its forests are currently a net source of emissions due to natural disasters and bark beetle infestations. The city's governance framework is exemplary, with a clear commitment to enhancing carbon sequestration through green spatial planning, urban agriculture, sustainable forest management as well as introducing mandatory green roofs and a pilot communal irrigation system. The governance framework includes the involvement of key stakeholders, such as the Slovenia Forest Service and the Institute for Agriculture, and the city has established a Green Spatial Plan to protect sub-urban forests and promote sustainable forest management.

Spain (2 Best Practices / 7 Mission Cities)

Barcelona

Barcelona's carbon sink governance stands out in that it prioritises the health and resilience of urban trees to maximise their social, economic, and environmental functions. The city focuses on creating a diverse and adaptable tree stock, with no single species dominating the urban landscape, to ensure a robust and thriving ecosystem. By promoting healthy trees, the city aims to enhance their role in mitigating climate change, while also fostering social interaction and community engagement with urban nature. The plan recognises the importance of trees in providing social and economic services, such as improving air quality, reducing urban heat islands, and supporting public health and wellbeing. By engaging citizens and promoting a culture of appreciation for urban trees, Barcelona's Tree Master Plan sets a model for effective carbon sink governance that balances environmental, social, and economic benefits.

The city complements its strategy with carbon sequestration in wooden buildings, urban agriculture, and blue carbon projects (Posidonia).

Zaragoza

Zaragoza intends to create a strategy to map the city's green spaces, forests, and tree populations. This initiative aims to leverage remote sensing technology to assess their capacity to absorb CO2. By doing so, the council can strategically plan the expansion of its green areas to sequester 5 to 7% of Zaragoza's total carbon emissions. The plan involves employing cutting-edge technologies to analyse, construct, and track carbon sequestration and other ecosystem metrics. This includes the use and analysis of high-resolution, multispectral satellite imagery to serve as a basis for analytical work. The initiative also considers the immediate application of precision satellite technology for the automated cataloguing of species and features related to green zones. Additionally, the project will develop land observation indices (in line with the European USAGE project – Urban Data Space for Green Deal) to assess vegetation health within the city, and to identify and characterise urban heat islands, which will be marked as priority areas for action. They comprise a total of 26 ecosystemic indicators represented in 150x150 m grids and accompanied by a graphic representation by neighbourhood, in what are called “ecosystemic wheels”. Another project (VERSAT) targets the characterisation,

adaptation and implementation of an environment for the analysis and interpretation of multispectral satellite images (16 bands) of very high spatial resolution (15 cm/pixel - 30 cm/px - 50 cm/px) for their complementary use in the construction, elaboration and monitoring of ecosystem indicators. Zaragoza complements its green strategy with ecologisation and renaturalisation of its river area.

Sweden (3 Best Practices / 7 Mission Cities)

Stockholm

Stockholm aims to become climate negative by 2030. Following a pilot phase, a large-scale BECCS facility linked to a biofuel-fired combined heat and power plant is anticipated to be operational by 2025/2026, with the capacity to sequester 800 ktCO2 annually. The intention is to create a world-class BECCS facility, combining CO2 capture with heat recovery. This project is also intended to accelerate the development of a new market for net carbon removals. The BECCS Stockholm project has the potential to lead many other CCS projects to follow. The financing will rely on three sources: EU, national government, and the sales of negative emission certificates on a voluntary carbon market. The EU Innovation Fund has already granted €1.1 billion to the project. A portion of the negative emissions will be financed through sales to private entities, necessitating a system to prevent double counting. A system for bookkeeping and cancellation of carbon removal units is proposed to ensure that double claiming, i.e. that several companies (private operators) claim the same removal unit, cannot take place. Even though the capacity of the BECCS facility would remove more than what would be strictly needed to achieve net-zero emissions, the city is explicitly committed to continue efforts to further reduce its emissions. The city is also leveraging biochar produced from repurposed garden waste to enrich the soil in urban gardens and street-side tree plantings, thus boosting the growth of trees and shrubs. The surplus heat generated during the production of biochar is fed into the district heating network, further contributing to lower residual emissions. The city has performed a mapping of the carbon stock and carbon sinks in forests and in soil and will investigate methods for increasing the green space carbon stock in urban development processes. Further, the city is supporting urban farming through a business collaboration promoting digital advice for urban cultivation and innovative installations of climate protected cultivation techniques in urban and vacant industrial land.

Helsingborg

Helsingborg's approach to carbon sequestration encompasses a wide range of measures with a focus on technological and durable sinks. It includes the implementation of a BECCS facility at the cogeneration plant, the use of DACCS, the production of biochar, and the restoration and creation of forest land and shelterbelt plantations. Additionally, Helsingborg is promoting changed agricultural practices on agricultural land, rewetting of organogenic soils, and the increase of seaweed and eelgrass meadows. The city is also preserving and developing existing carbon sinks in sea, soil, and vegetation, and creating new parks and green areas.

Helsingborg's comprehensive strategy to address carbon sequestration is underpinned by solid quantification and scenario analysis to fully compensate for all residual emissions by 2030.

The analysis has identified a range of options for reducing emissions and increasing carbon sinks. The city has quantified its carbon sink potential, with an estimated total carbon sink of approximately 124,000 tCO_2 (100,000 from technological sinks, the rest from biochar and natural sinks). To close the gap to climate neutrality, Helsingborg is considering a range of alternatives, including the reduction of plastic in residual waste to reduce emissions and enhance the CCS potential, increased carbon sequestration in agricultural land, and increased forest plantation.

The city's governance framework is supportive of its carbon sequestration efforts, with a range of initiatives and instruments in place to promote sustainable land use, urban planning, and community engagement. Helsingborg has established a competence centre for biochar, and is working to expand production and encourage the use of biochar in agriculture and other sectors. The city is also engaging with private landowners and farmers to promote changed agricultural practices and the protection of natural land. Additionally, Helsingborg is using a range of tools and instruments, including the Climate OS tool, to monitor and evaluate its progress towards climate neutrality.

Helsingborg's monitoring protocol is designed to track the effectiveness of its carbon sequestration efforts and identify areas for improvement. The city is using a range of KPIs, including carbon capture and storage at the district heating plant, changed agricultural practices on agricultural land, and the amount of permanent sequestration of GHG within the city boundary. The city is also monitoring the operation of its new biochar plant, the creation of new parks and green areas, and the restoration of natural land. Overall, Helsingborg's approach to carbon sequestration is a best practice, due to its comprehensive and quantitative approach, its variety of options, and its commitment to monitoring and evaluation.

Malmö

The city's Action Plan outlines a wide range of compensation mechanisms, such as green infrastructure, soil carbon sequestration, biochar, biomass in construction, BECCS, and biochar. Additionally, Malmö is exploring the use of carbon credits through pilot projects on purchasing negative emissions. Malmö's strategy is robust and well-researched, with studies demonstrating the potential for the city to become climate negative. The city is experimenting with biochar in various municipal applications and has partnered with the main biochar innovation and development project in the country. Furthermore, BECCS is considered for both the waste-to-energy plant and district heating. The city's approach to carbon sequestration is diverse, considering both temporary and permanent sinks, and includes compelling quantification and governance mechanisms to ensure effective implementation.

Türkiye (1 Best Practice / 2 Mission Cities)

Izmir

Izmir proposes a comprehensive approach to land use for carbon sequestration. Its strategy addresses urban greening, sustainable agriculture, and forestry while promoting sustainable land use and agriculture practices. Its low-carbon farming strategy focuses on soil and

grassland management along with nutrient and livestock management to optimise carbon sequestration, along with precision agriculture and renewables to maximise the net negative emissions effect. The strategy also insists on community engagement and education with the formation of farmer cooperatives, farmer advisory groups, and awareness campaigns to help educate farmers on climate-smart agriculture practices and promote the adoption of sustainable methods. Finally, the establishment of a dedicated department for monitoring and enforcing climate-smart practices in agriculture ensures that the strategy's goals are tracked. The city also looks at a multipurpose agenda. For instance, the creation of Living Parks and İzmir Heritage Routes is meant to protect biodiversity and promote ecosystem services.

United Kingdom (1 Best Practice / 2 Mission Cities)

Glasgow

The city's measures include afforestation and reforestation, greening and rewilding, tree planting, effective use of vacant and derelict land sites, creation of new local nature reserves, street trees, green roofs, sustainable harvested timber, urban agriculture, and wetlands restoration, including peatlands. The city promotes a range of initiatives and instruments to support its carbon sequestration efforts. The Ecological Emergency Plan will be implemented via the Open Space Strategy Delivery Plan, which will map out opportunities to enhance Glasgow's active travel routes and green network linkages. The city has also developed a local biodiversity action plan and open space strategy delivery plan, which will investigate opportunities to increase and protect biodiversity. The creation of new Local Nature Reserves will complement the enhanced network of open spaces across the city, helping to mitigate the urban heat island effect. The city will take forward 30+ aspirational Local Nature Reserves and increase investment in peatland restoration in the city region to enhance biodiversity and increase capacity for carbon sequestration. The city will develop engagement with all local and regional stakeholders, including landowners, to ensure that Glasgow's peatlands are restored and protected. The city is also developing an Urban Woodland Strategy, which will look at how trees deliver a range of benefits, including carbon capture, development, leisure, heat reduction, water management, food, biodiversity, and construction. An environmental digital model is being developed alongside this strategy. The city is also accelerating the implementation of the Glasgow and Clyde Valley Blueprint for a Green Network, which will create green and biodiversity corridors in the city by improving open space provision and increasing tree cover. The city's initiatives, such as the Glasgow Clyde Valley Green Network Partnership, Sustainable Glasgow Partnership, and Climate Ready Clyde, create an enabling environment for the deployment of nature-based solutions and support the development of actions in the agriculture, forestry, and other land use sector. These partnerships and collaborations facilitate the development of regional approaches to increasing carbon sequestration and biodiversity gains, while also promoting the restoration of peatland, wetland, woodland, and grassland. The city is also developing a Peat Free Procurement Strategy, which will require 100% peat-free products for specific projects, such as nursery and bedding plants. The city will engage with procurement units and suppliers to establish the best alternative products available and develop a peat-free procurement strategy.
