# Supplementary material for: Empirical insights into residual emissions in urban net-zero targets
Source: Nat Clim Chang. 2026 Aug 14;16(9):1092–101. doi: 10.1038/s41558-026-02691-0 (PMC13538053; doi:10.1038/s41558-026-02691-0)
Supplement: Supplementary file 2 [file 41558_2026_2691_MOESM2_ESM.pdf]

nature portfolio

Corresponding author(s): Giulia Ulpiani

Last updated by author(s): Feb 28, 2026

Reporting Summary

Nature Portfolio wishes to improve the reproducibility of the work that we publish. This form provides structure for consistency and transparency in reporting. For further information on Nature Portfolio policies, see our Editorial Policies and the Editorial Policy Checklist.

Statistics

For all statistical analyses, confirm that the following items are present in the figure legend, table legend, main text, or Methods section.

n/a	Confirmed	
☐	☒	The exact sample size (*n*) for each experimental group/condition, given as a discrete number and unit of measurement
☒	☐	A statement on whether measurements were taken from distinct samples or whether the same sample was measured repeatedly
☐	☒	The statistical test(s) used AND whether they are one- or two-sided<br>*Only common tests should be described solely by name; describe more complex techniques in the Methods section.*
☐	☒	A description of all covariates tested
☐	☒	A description of any assumptions or corrections, such as tests of normality and adjustment for multiple comparisons
☐	☒	A full description of the statistical parameters including central tendency (e.g. means) or other basic estimates (e.g. regression coefficient) AND variation (e.g. standard deviation) or associated estimates of uncertainty (e.g. confidence intervals)
☒	☐	For null hypothesis testing, the test statistic (e.g. *F*, *t*, *r*) with confidence intervals, effect sizes, degrees of freedom and *P* value noted<br>*Give P values as exact values whenever suitable.*
☒	☐	For Bayesian analysis, information on the choice of priors and Markov chain Monte Carlo settings
☒	☐	For hierarchical and complex designs, identification of the appropriate level for tests and full reporting of outcomes
☒	☐	Estimates of effect sizes (e.g. Cohen's *d*, Pearson's *r*), indicating how they were calculated

Our web collection on statistics for biologists contains articles on many of the points above.

Software and code

Policy information about availability of computer code

Data collection	The analysis is based on the action plans that cities submitted as part of the 100 Climate Neutral and Smart Cities Mission. The plans are publicly available at https://netzerocities.app/knowledge-ccc, as indicated in the manuscript.
Data analysis	The analysis is based on an Excel sheet where we collected the results of our coding. The results were then analysed using Python (version 3.11) for simple statistics and illustrations.

For manuscripts utilizing custom algorithms or software that are central to the research but not yet described in published literature, software must be made available to editors and reviewers. We strongly encourage code deposition in a community repository (e.g. GitHub). See the Nature Portfolio guidelines for submitting code & software for further information.

Data

Policy information about availability of data

All manuscripts must include a data availability statement. This statement should provide the following information, where applicable:

- Accession codes, unique identifiers, or web links for publicly available datasets
- A description of any restrictions on data availability
- For clinical datasets or third party data, please ensure that the statement adheres to our policy

The dataset that underpins the analysis of the emissions compensation strategies is provided as Supplementary Data.

Research involving human participants, their data, or biological material

Policy information about studies with human participants or human data. See also policy information about sex, gender (identity/presentation), and sexual orientation and race, ethnicity and racism.

Reporting on sex and gender	Not applicable
Reporting on race, ethnicity, or other socially relevant groupings	Not applicable
Population characteristics	Not applicable
Recruitment	Not applicable
Ethics oversight	Not applicable

Note that full information on the approval of the study protocol must also be provided in the manuscript.

Field-specific reporting

Please select the one below that is the best fit for your research. If you are not sure, read the appropriate sections before making your selection.

☐ Life sciences ☐ Behavioural & social sciences ☒ Ecological, evolutionary & environmental sciences

For a reference copy of the document with all sections, see nature.com/documents/nr-reporting-summary-flat.pdf

Ecological, evolutionary & environmental sciences study design

All studies must disclose on these points even when the disclosure is negative.

Study description	The study presents a comprehensive and quantified analysis of urban residual emissions and compensation strategies across 103 cities, covering not only the technical aspects of residual emissions but also the overall strategy for compensation. We examine cities' capacity to determine the origin of residual emissions, the carbon sequestration potential of different options, the timing for implementation, the attention to monitoring, reporting, and verification provisions, as well as the governance structures, planning instruments and incentives that support these efforts, all in a quantitative manner. The analysis culminates in a 2030-2050 projection that condenses evidence and practical recommendations. Further, the paper is accompanied by a rich Supplementary Material that outlines best practices, serving as a valuable resource for cities, urban practitioners, and scholars.
Research sample	The analysis is based on the climate neutrality action plans submitted by a total of 103 cities (two of which in a joint plan) in four consecutive windows of submission between 2023 and 2025. Urban administrations are the unit of analysis. Regionally, the analysis is focused on Europe. The city sample encompasses cities of 17 thousand inhabitants as well as large conurbations of up to 17.8 million inhabitants (median=319 thousand inhabitants), covering 30 countries. The greatest representation (>5 cities) is of Italian, French, Spanish, Swedish, German, Finnish, and Greek cities, in this order. Numerous governance arrangements are included, which may manifest as different degrees of maturity and integration of carbon dioxide removals and carbon credits in urban climate action. The sample includes a good balance between coastal and inland cities (41.2% versus 58.8%) and a variety of climatic conditions (all five main Köppen-Geiger classes but tropical climates).
Sampling strategy	We focus on the cities that were selected in the 100 Climate Neutral and Smart Cities Mission. In total 113 cities have been selected. The 103 Mission Cities we analyse are those that received the European Commission's Mission Label as of October 2025, with the Label standing as an acknowledgement of the successful completion of the process followed by cities to develop their Climate City Contracts, formed by Commitment, Action and Investment Plans. Labelled cities upload their plans in a public repository as indicated above. Nine Mission Cities do not feature in this analysis as they are expected to submit their plans by 2026.
Data collection	To generate comprehensive and comparable information, this analysis relies on a labour-intensive, comprehensive process of individually reviewing and coding information across the action plans. A team of 61 trained experts and data collectors was involved in the analysis of the plans. Through content analysis (both conceptual and relational analysis), coders conducted a systematic manual scrutiny of the plans (texts, images and symbolic matter) to gather evidence. The strategies to compensate for unabated emissions were coded based on a carefully crafted classification, based on recent guidance and regulations (e.g., the Carbon Removal and Carbon Farming Regulation in Europe). The robustness of the residual emissions approach and strategy is measured against 6 main macro-dimensions concerning aspects of quantification, strategic mapping, timing, monitoring, and governance. A number of general city attributes (e.g., climatic class, coastal versus inland city, GDP per capita, presence of ports, airports within the geographic boundary and in a 10km radius, land cover from the Corine dataset, and land use efficiency from the Global Human Settlement Layer) complement the data collection, via GIS retrieval (all sources are specified in the manuscript). Finally, data on national climate strategies on carbon dioxide geological storage was retrieved from a stocktake by Alcalde et al. (reference in the manuscript) looking into the presence and prevalence of different options for CCS and subsurface CDR in national decarbonisation pathways worldwide.
Timing and spatial scale	We started coding the data in April 2023. Each plan was coded once. The plans refer to 103 European cities.

Data exclusions	No exclusions
Reproducibility	There is no experiment involved. The actions plans of the cities can be analysed using the method described in the paper.
Randomization	Not applicable
Blinding	Not applicable

Did the study involve field work? ☐ Yes ☒ No

Reporting for specific materials, systems and methods

We require information from authors about some types of materials, experimental systems and methods used in many studies. Here, indicate whether each material, system or method listed is relevant to your study. If you are not sure if a list item applies to your research, read the appropriate section before selecting a response.

Materials & experimental systems

n/a	Involved in the study
☒	☐ Antibodies
☒	☐ Eukaryotic cell lines
☒	☐ Palaeontology and archaeology
☒	☐ Animals and other organisms
☒	☐ Clinical data
☒	☐ Dual use research of concern
☒	☐ Plants

Methods

n/a	Involved in the study
☒	☐ ChIP-seq
☒	☐ Flow cytometry
☒	☐ MRI-based neuroimaging

Plants

Seed stocks	Not applicable
Novel plant genotypes	Not applicable
Authentication	Not applicable
