# Supplementary material for: Empirical insights into residual emissions in urban net-zero targets
Source: Nat Clim Chang. 2026 Aug 14;16(9):1092–101. doi: 10.1038/s41558-026-02691-0 (PMC13538053; doi:10.1038/s41558-026-02691-0)
Supplement: Supplementary file 3 [file 41558_2026_2691_MOESM3_ESM.pdf]

**Display item number**	**Rights holder**	**Reference and source**, *if applicable.*
Fig. 2 & Fig. 4	freepik	"Assortment of green trees in isometric style" https://www.freepik.com/free-vector/assortment-green-trees-isometric-style_1060682.htm#fromView=search&page=1&position=7&uuid=723f378d-b5fe-4a4a-9fbe-716d5a58ec6a&query=isometric+tree
Fig. 2	macrovector	"Set of ground surfaces. Grass, rocks and water. Landscape web design. Vector illustration" https://www.freepik.com/free-vector/set-ground-surfaces-grass-rocks-water-landscape-web-design-vector-illustration_11059308.htm#fromView=search&page=1&position=28&uuid=b328abfe-ce0e-4b76-9c02-138a0ea128f2&query=isometric+grass
Fig. 2	macrovector	"Set with isolated modern agricultural machinery isometric icons with combine harvesters heavy tractors and plow bulldozers vector illustration" https://www.freepik.com/free-vector/set-with-isolated-modern-agricultural-machinery-isometric-icons-with-combine-harvesters-heavy-tractors-plow-bulldozers-vector-illustration_39221817.htm#fromView=search&page=1&position=9&uuid=0aa7bef2-35b1-46ee-9725-e931cb3a0b44&query=isometric+tractor
Fig. 2	macrovector	"Ecology isometric icons set on theme of green energy clean planet and eco home isolated on white background vector illustration" https://www.freepik.com/free-vector/ecology-isometric-icons-set-theme-green-energy-clean-planet-eco-home-isolated-white-background-vector-illustration_41922734.htm#fromView=search&page=1&position=39&uuid=791a43fd-6f92-4b90-95f0-17411dd2f167&query=isometric+bioenergy
Fig. 2 & Fig. 4	macrovector	"Park element set with plants and trees isometric isolated vector illustration" https://www.freepik.com/free-vector/park-element-set-with-plants-trees-isometric-isolated-vector-illustration_26765352.htm#fromView=search&page=1&position=25&uuid=196c7ef0-4ea3-425c-a148-535e78150c0a&query=isometric+grass
Fig. 2	macrovector	"Isometric icons set with various trees plants flowers and decorations for botanical garden isolated on white 3d" https://www.freepik.com/free-vector/isometric-icons-set-with-various-trees-plants-flowers-decorations-botanical-garden-isolated-white-3d_6845816.htm#fromView=search&page=1&position=1&uuid=a1457d49-3c5a-40e8-98eb-0dd8bccf5a74&query=isometric+water+plants
Fig. 2	macrovector	"Landscaping set of isometric icons with elements green lawn trees and bushes isolated" https://www.freepik.com/free-vector/landscaping-set-isometric-icons-with-elements-green-lawn-trees-bushes-isolated_7286346.htm#fromView=search&page=1&position=12&uuid=c7c00e3e-dd43-40ae-855d-c5a58d33acdc&query=isometric+bushes
Fig. 2 & Fig. 4	macrovector	"Professional gardening isometric icons with elements of landscape design and people working outdoor isolated vector illustration" https://www.freepik.com/free-vector/professional-gardening-isometric-icons-with-elements-landscape-design-people-working-outdoor-isolated-vector-illustration_37581767.htm#fromView=search&page=1&position=7&uuid=c7c00e3e-dd43-40ae-855d-c5a58d33acdc&query=isometric+bushes
Fig. 2	studiogstock	"Pile coins money dollars icons" https://www.freepik.com/free-vector/pile-coins-money-dollars-icons_169504318.htm#fromView=search&page=1&position=0&uuid=c746cacd-cdc8-4d64-a1b1-17fdde0bbc5c&query=coins
Fig. 2 & Fig. 4	macrovector	"Carbon capture storage sequestration technology infographics with icons of offshore storage factory transportation methods and text vector illustration" https://www.freepik.com/free-vector/carbon-capture-storage-sequestration-technology-infographics-with-icons-offshore-storage-factory-transportation-methods-text-vector-illustration_41922737.htm#from_element=cross_selling__vector
Fig. 2 & Fig. 4	macrovector	"Design groups of rocks and stones boulders" https://www.freepik.com/free-vector/design-groups-rocks-stones-boulders_3792126.htm#fromView=search&page=1&position=42&uuid=484b934c-5e3e-4c47-9424-40693fea4f6c&query=isometric+black+rock
Fig. 4	macrovector	"House construction icons set" https://www.freepik.com/free-vector/house-construction-icons-set_4342029.htm#fromView=search&page=1&position=0&uuid=75db540d-6dea-4aa5-abc7-2435e3ecd7c1&query=wood+building+isometric
Fig. 2 & Fig. 4	valadzionak_volha	"Marsh reed grass stone in pond set of swamp cattails rock in lake vector bulrush" https://www.freepik.com/free-vector/marsh-reed-grass-stone-pond-set-swamp-cattails-rock-lake-vector-bulrush_26984545.htm#fromView=search&page=1&position=7&uuid=dc8e50b9-9706-4a45-9f2b-0f27b7cfdc97&query=isometric+water+plants
Fig. 2	macrovector	"Building industry isometric" https://www.freepik.com/free-vector/building-industry-isometric_3815781.htm#from_element=cross_selling__vector

Fig. 2	macrovector	"Isometric jungle set with isolated elements of jungle terrain with trees and plants on blank background vector illustration" https://www.freepik.com/free-vector/isometric-jungle-set-with-isolated-elements-jungle-terrain-with-trees-plants-blank-background-vector-illustration_26760436.htm#fromView=search&page=1&position=3&uuid=4cf7fa27-d07a-49de-a8f9-0719ba603277&query=isometric+jungle+tree
Fig. 2, Fig. 3 & Fig. S1	Natural Earth	Natural Earth – Free vector and raster map data at 1:10m, 1:50m, and 1:110m scales. https://www.naturalearthdata.com/

All other icons in Fig. 4 (including: factory building, truck, building, car, utility pole, plug, lightning, plane, crane, downward graph, magnifying lens, balance, check list, document search, and dialogue) are from Microsoft PowerPoint 365.
